# *Enterococcus faecium* TIR-Domain Genes Are Part of a Gene Cluster Which Promotes Bacterial Survival in Blood

**DOI:** 10.1155/2018/1435820

**Published:** 2018-12-03

**Authors:** Theresa M. Wagner, Jessin Janice, Fernanda L. Paganelli, Rob J. Willems, Fatemeh Askarian, Torunn Pedersen, Janetta Top, Carla de Haas, Jos A. van Strijp, Mona Johannessen, Kristin Hegstad

**Affiliations:** ^1^Research Group for Host-Microbe Interactions, Department of Medical Biology, Faculty of Health Sciences, UiT-The Arctic University of Norway, Tromsø, Norway; ^2^Norwegian National Advisory Unit on Detection of Antimicrobial Resistance, Department of Microbiology and Infection Control, University Hospital of North-Norway, Tromsø, Norway; ^3^Department of Medical Microbiology, University Medical Center Utrecht, Utrecht University, Utrecht, Netherlands

## Abstract

*Enterococcus faecium* has undergone a transition to a multidrug-resistant nosocomial pathogen. The population structure of *E. faecium* is characterized by a sharp distinction of clades, where the hospital-adapted lineage is primarily responsible for bacteremia. So far, factors that were identified in hospital-adapted strains and that promoted pathogenesis of nosocomial *E. faecium* mainly play a role in adherence and biofilm production, while less is known about factors contributing to survival in blood. This study identified a gene cluster, which includes genes encoding bacterial Toll/interleukin-1 receptor- (TIR-) domain-containing proteins (TirEs). The cluster was found to be unique to nosocomial strains and to be located on a putative mobile genetic element of phage origin. The three genes within the cluster appeared to be expressed as an operon. Expression was detected in bacterial culture media and in the presence of human blood. TirEs are released into the bacterial supernatant, and TirE2 is associated with membrane vesicles. Furthermore, the *tirE*-gene cluster promotes bacterial proliferation in human blood, indicating that TirE may contribute to the pathogenesis of bacteremia.

## 1. Introduction

In the last three decades, the Gram-positive bacterium *Enterococcus* has undergone a pronounced transition from a human gut commensal to a multidrug-resistant nosocomial opportunistic pathogen [1–3]. Enterococcal infections are mainly caused by *Enterococcus faecium* and *E. faecalis* [4, 5]. *E. faecalis* historically accounted for 80–90% of the clinical isolates, and 5–10% used to be *E. faecium* [6]. However, *E. faecium* has risen to be the cause of 34% of the enterococcal infections [7].

The clade structure of *E. faecium* displays distinct groups, where commensal strains fall into clade B and hospital-associated strains into clade A [8, 9]. Enterococcal genomes are highly plastic and are able to acquire and exchange large fragments of DNA. Especially in clade A, the cumulative adaptive gene uptake is reflected by a larger genome size [8].

The most severe infection caused by *E. faecium* is endocarditis, and additionally, *E. faecium* often causes urinary tract infection and bacteremia [4]. Infections are established upon translocation to the bloodstream, through the perturbed intestine or uptake from the contaminated hospital environment or skin [4].

The host innate immune system provides the first line of defense against microbial attacks. It is induced through the recognition of highly conserved microbial structures, namely, pathogen-associated molecular patterns (PAMPs), by pattern recognition receptors, among which Toll-like receptors (TLRs) are predominant [10, 11]. The recognition of PAMPs by TLRs mediates antimicrobial responses, such as phagocytosis and microbial killing. Out of ten presently described human TLRs, TLR2 has been identified to be central in recognition of *E. faecium* [12]. MyD88 is the key intracellular adaptor for TLR2, and stimulation of its intracellular pathway leads to NF-kB activation, resulting in proinflammatory mediator expression [13–15]. Since the initial recognition of a pathogen by immune cells is crucial for all subsequent antimicrobial responses of the host, interference with the innate immune response is a significant advantage for invasive bacteria, and counteraction is through molecular mimicry and immune evasion factors [11, 16].

Bacterial TIR- (Toll/interleukin-1 receptor-) domain-containing proteins structurally mimic host domains, which are crucial for protein-protein interaction in TLR signaling cascade adaptors [17, 18]. Immune evasive properties of bacterial TIR proteins were first described in *Salmonella enterica*, where the protein was named “TlpA” (TIR-like protein A) [18]. Since then, TIR proteins with immune evasive properties have been described in a range of Gram-negative and Gram-positive bacteria, including *Staphylococcus aureus* [19, 20] and *E. faecalis* [21]. In general, bacterial TIR proteins negatively interfere with TLR signaling through adaptor blockade or adaptor degradation [22]. TIR-domain-containing proteins share several amino acid sequence motifs, such as boxes 1 and 2 as well as the WxxxE motif [22, 23]. The TIR-domain-containing protein in *E. faecalis* (TcpF) has been described to be predominant among urinary tract infection isolates, attenuate MyD88-mediated signaling, and promote bacterial survival within macrophages [21, 24]. In the closely related *E. faecium*, however, TIR-domain-containing proteins have not been described yet. The virulence factors so far identified in *E. faecium* are mainly associated with adhesion, aggregation, growth, and biofilm formation [25–42]. Identification of virulence factors might reveal targets for novel therapeutic approaches.

Our aims were to identify TIR-domain-containing proteins in *E. faecium* (TirEs), compare their prevalence in nosocomial versus community-associated strains, explore their genetic context, and study their function.

## 2. Materials and Methods

### 2.1. Bacterial Strains, Plasmids, and Growth Conditions

Prevalence screening for *tirE* genes and the gene *hp1* between the two *tirE* genes was performed in a diverse *E. faecium* collection. The strains were of different geographical origin (24 countries), the main countries being the Netherlands (587 isolates), Latvia (96 isolates), Norway (88 isolates), Switzerland (82 isolates), Greece (79 isolates), Germany (61 isolates), Portugal (56 isolates), and Denmark (40 isolates). The total of 1194 strains consist of blood culture (161 isolates), other hospital-associated isolates (meaning feces, urine, or wound, 875 isolates), and human community (158 isolates) isolates (Table S1).

*E. faecium* and *Escherichia coli* strains as well as plasmids used for laboratory experiments in this study are listed in Table S2. *E. faecium* was grown in the brain heart infusion (BHI) medium or Luria-Bertani (LB) medium at 30°C or 37°C with shaking; *E. coli* strains were grown in the LB medium or LB supplemented with 20 mM glucose at 37°C with shaking.

For *E. faecium*, the antibiotics gentamicin and spectinomycin were used at a concentration of 300 *µ*g/ml and erythromycin at 25 *µ*g/ml. For *E. coli*, gentamicin and spectinomycin were used at a concentration of 30 *µ*g/ml and 100 *µ*g/ml, respectively.

For the Rosetta-gami (DE3) pLysS *E. coli* protein expression strain, the following antibiotic concentrations were used: ampicillin 100 *µ*g/ml, chloramphenicol 50 *µ*g/ml, tetracycline 12.5 *µ*g/ml, kanamycin 15 *µ*g/ml, and streptomycin 50 *µ*g/ml. All antibiotics were obtained from Sigma-Aldrich (USA).

### 2.2. Bioinformatic Analysis of TIR-Domain-Containing Proteins

Putative genes encoding TIR-domain-containing proteins were identified in the *E. faecium* E1162 genome (GenBank: ABQJ00000000), and protein sequences were found in contig107 (ABQJ01000097.1) with locus tags EFME1162_RS19585 (old locus tag EfmE1162_2149) and EFME1162_RS19595 (old locus tag EfmE1162_2151), respectively.

TIR domains and protein families were verified through searches in the Conserved Domain Database (CDD) [43]. Alignment to previously described bacterial and eukaryotic TIR proteins (BtpA from *Brucella melitensis* [EXU84762.1], TcpC from *E. coli* [NP_754290], YpTdp from *Yersinia pestis* [WP_002213208.1], PdTir from *Paracoccus denitrificans* [WP_011746463.1], TirS from *S. aureus* [SAS0038, WP_000114516.1], SaTlp1 from *S. aureus* [CAQ50581.1], TcpF from *E. faecalis* [CCO72761.1], Myd88 from *Homo sapiens* [AAH13589.1], and TLR2 from *Homo sapiens* [AAC34133.1]) was performed in the multiple sequence alignment program MAFFT version 7 [44], and the alignment was viewed and manually curated in AliView [45]. The alignment was illustrated using ESPript 3.x with secondary structure elements of BtpA [46], accessed under 4lzp in the RCSB Protein Data Bank [47, 48]. A phylogenetic tree was constructed with the algorithm PROTGAMMAAUTO with 100 bootstrap replicates for these protein sequences using RAxML v8.2 [49]. The secondary structures were predicted in the protein fold recognition server Phyre2 [50], superposed to the crystal structure of the TIR domain of human MyD88 using PDBe Fold v2.59 [51], and illustrated in PyMOL [52].

### 2.3. Detection of Genes by PCR

PCR was performed with gene-specific primers (prevalence screening primers (Table S3)) and DreamTaq Green PCR Master Mix (Thermo Fischer Scientific, USA) according to the manufacturer's instruction. One microliter of bacterial culture in BHI broth was used as the template. A standard program of 10 min initial denaturation at 95°C followed by 30 cycles of 30 s denaturation at 95°C, 30 s annealing at 55°C, and 30 s elongation at 72°C with a final elongation step for 7 min at 72°C was used. PCR products were thereafter visualized on a 1% agarose gel.

### 2.4. Phylogenomics

To study the distribution of *tirE* genes, *E. faecium* genome assemblies were downloaded on June 27, 2017 (*n* = 516), from the National Center for Biotechnology Information (NCBI). BLASTp [53, 54] searches were performed against all the downloaded genomes for the proteins TirE1 (EFF33949.1), Hp1 (EFF33950.1), and TirE2 (EFF33951.1). A core genome phylogenetic tree was constructed with all *E. faecium* genomes using Parsnp [55]. The resulting tree was visualized and annotated for the presence or absence of *tirE1*, *hp1*, and *tirE2* genes, in FigTree v1.4.3 [56]. Metadata including the country of origin, isolation source, disease information, assembly level, and antimicrobial-resistant phenotypes were collected for each of the *tirE*-positive genomes (Table S4) from NCBI and PATRIC [57].

### 2.5. Identification of a Putative Mobile Genetic Element

The genetic regions up- and downstream of the *tirE* locus in the *E. faecium* E1162 genome sequence were compared to the corresponding regions of the community strain *E. faecium* 17OM39, which lacks the *tirE* region. The comparison was viewed and analyzed using Artemis Comparison Tool [58]. Pairwise comparison figures were drawn using EasyFig 2.2.2 [59, 60]. Insertion of a putative mobile genetic element in E1162 was identified, and each gene in this region was subjected to BLAST [53] and CDD [43] searches to identify the similar proteins and protein domains and to Phyre2 [50] for structural, functional, and evolutionary classification prediction (Table 1).

The inserted genetic region that is flanked by phage integrases was extracted from all the *tirE*-locus-positive genomes. Genomes, which contained incomplete insertion elements, because of assembly incompleteness, were excluded from further analyses. The mobile genetic elements were reannotated in Prokka 1.11 [61], and proteins were clustered with Roary 3.8.2 [62]. To understand the conserved nature of these elements, a heat map was created and visualized in GI tools [63, 64].

### 2.6. mRNA Expression Analysis by Reverse Transcription (RT) PCR

For blood exposure, bacteria (E1162 and K60-39) were grown in BHI to OD_600_ 0.4, washed with PBS, and resuspended in the Roswell Park Memorial Institute (RPMI) medium with 0.05% human serum albumin (RPMI-HSA). Fresh human blood was collected from healthy volunteers in hirudin blood tubes (Roche Diagnostics, Switzerland), added to the bacteria to a final concentration of 80%, and incubated on a turning wheel at 37°C for 3 h. Blood cells were lysed in 0.3% ice-cold saponin, and the bacterial pellet was washed with PBS. All bacterial samples were stored in RNAprotect solution (Qiagen, Germany). RNA was extracted using the RNeasy Mini Kit (Qiagen, Germany) following the manufacturer's instructions with a prolonged initial lysis step using mutanolysin (0.1 U/*µ*l) and lysozyme (1 mg/ml) for 1 h at 37°C. After DNase treatment (Heat and Run Kit; ArcticZymes, Norway), RNA integrity and quantity were checked by NanoDrop as well as on agarose gel. Reverse transcription was performed (High-Capacity cDNA Reverse Transcription Kit; Applied Biosystems, USA) on 100 ng RNA, and DNA contamination was ruled out through minus reverse transcriptase (−RT) control. The genes were thereafter amplified from the cDNA through PCR (prevalence screening primers (Table S3); 30 cycles; annealing temperature 55°C), and the products were visualized on a 1% agarose gel.

In order to assess whether the genes are expressed together, junction primers were used on the cDNA template to amplify the intergenic regions (junction primers (Table S3); 30 cycles; annealing temperature 55°C). Additionally, the internal primers (prevalence screening primers (Table S3); 30 cycles; annealing temperature 55°C) were combined to link the gene product. The identity of gene products was confirmed by sequencing the PCR products.

### 2.7. Cloning, Expression, and Purification of Recombinant N-His-TirE

The E1162 *tirE1*, *hp1*, and *tirE2* genes were amplified with BamHI-Fw and NotI-Rv primers (expression primers (Table S3)) and cloned in frame into the expression plasmid pRSETB containing an N-terminal HIS_6_ tag (Invitrogen, USA) (Table S3), as previously described [65]. After verification of the correct sequence by DNA sequencing, the pRSET/TirE1, pRSET/Hp1, and pRSET/TirE2 constructs were transformed into *E. coli* Rosetta-gami (DE3) plysS. Expression was induced in the mid-logarithmic growth phase with 1 mM isopropyl *β*-d-1-thiogalactopyranoside (IPTG) for 4 h or overnight (Figure S1A).

Proteins were isolated from an HiTrap Chelating HP column under either native (Figure S1B) or denaturing (8 M urea (Figure S1C)) conditions and eluted using an imidazole gradient, ranging from 10 to 500 mM in an ÄKTA FPLC protein purification system (GE Healthcare Life Sciences, Australia). The protein-containing fractions were pooled. Fractions containing denaturated proteins were refolded through dialysis with salt buffer (50 mM Tris and 30 mM NaCl, pH 7.8). Finally, proteins from both native and denaturating conditions were dialyzed with PBS. The purity of the proteins was confirmed by SDS-PAGE. The calculated protein sizes with the his tag were TirE1 20.4 kDa, TirE2 34.9 kDa, and Hp1 56.9 kDa.

### 2.8. Anti-TirE Serum, Immunoprecipitation, and Western Blot

Purified TirE1, Hp1, and TirE2 were then used to immunize *Enterococcus*-negative rabbits by Eurogentec (Belgium). Preimmune serum was tested for nonreactivity towards enterococcal lysates prior to injection.

Bacteria (E1162 and K60-39) from 25 ml overnight culture were pelleted and resuspended in the lysis buffer (lysozyme 10 mg/ml and mutanolysin 6 U/*µ*l), incubated for 1 h at 37°C, and sonicated (4 times 1 min on-off). The bacterial supernatant was concentrated 40 times by centrifugation using a 10 kDa cutoff filter and sonicated (4 times 1 min on-off). Immunoprecipitation was performed using SureBeads Protein G magnetic beads (Bio-Rad, USA) according to the manufacturer. In brief, the beads were washed with PBS-T (PBS with 0.01% Tween), and 50% anti-TirE1, anti-TirE2, or anti-Hp1 serum in PBS was added. After washing three times, the bacterial lysate or supernatant was incubated with the beads for 1 h at room temperature under shaking. Finally, the samples were eluted into 15 *µ*l western blot sample buffer.

Western blot was performed as described previously [66]. For immunodetection, the polyclonal antiserum against TirE1 (1 : 300), Hp1 (1 : 300), and TirE2 (1 : 300) was used. Swine horseradish peroxidase- (HRP-) conjugated anti-rabbit IgG antibodies (1 : 3000) were used as secondary antibodies.

### 2.9. Membrane Vesicle Isolation from *Enterococcus*

Membrane vesicles were isolated from *E. faecium* as described earlier [67]. In short, bacteria (K60-39 and DO) were grown to the stationary phase in LB, and bacterial cells were removed by spinning down the culture for 30 min at 4°C at 6000 g. The supernatant was filtrated through 0.22 *µ*m vacuum-bottle top filters (Millipore, USA). A vesicular pellet was then obtained from the supernatant through ultracentrifugation at 4°C at 100.000 g for 4 h, washed with PBS, and centrifuged again at 4°C at 100.000 g for 4 h. The vesicular pellet was thereafter cleaned through density gradient centrifugation using OptiPrep (Sigma-Aldrich, USA), where the MV-containing samples were bottom loaded. 200 *µ*l fractions were analyzed in SDS-PAGE, and vesicle-containing fractions were selected. Proteins were TCA precipitated from the respective fractions and analyzed in a Thermo Scientific Q-Exactive mass spectrometer (Thermo Fisher Scientific, USA) after solution trypsinization. The raw data were processed in Proteome Discoverer 2.1 software. The fragmentation spectra were searched against WGS data of the strain itself (*E. faecium* DO and K60-39) using the Sequest HT program.

### 2.10. Markerless Mutant Construction

Utilizing the Cre-*lox* recombination system, markerless gene deletion mutants in the *tirE* gene cluster (locus_tag: EFME1162_RS19585 to EFME1162_RS19595) were created as previously described [40, 68, 69]. In short, gblock (TirE1_up-Tir2_down (Table S3)) consisting of the 5′ and 3′ flanking regions (approximately 500 bp each) of the target with an *Eco*RI site between both fragments was amplified with tirE1_up and tirE2_down primers (Table S3) and cloned into the vector pWS3 [70], resulting in pDELtir. Then, a gentamicin-resistant cassette flanked by *lox66* and *lox71* sites [69] was cloned into the *Eco*RI site included in the TirE1_up-Tir2_down gblock between the 5′ and 3′ flanking regions in pDELtir.

The resulting plasmid pDELtirGenta was subsequently electroporated in competent *E. faecium* E1162, as previously described. Marked mutants were obtained by growing the gentamicin-resistant transformants at appropriate temperatures supplemented with respective antibiotics. The plasmid pWS3-Cre carrying a gene encoding the Cre recombinase was introduced into the marked mutant by electroporation. Subsequent culturing to remove the gentamicin-resistant cassette and thereby ensure loss of pWS-Cre was performed as previously described [69]. Excision of the gentamicin-resistant cassette and loss of pWS3-Cre were verified by PCR using the primers tirE1-check-up and tirE1-check-down, in RT-qPCR using internal primers (prevalence screening primers (Table S3)) as well as through whole-genome sequencing (WGS) which also ensured that no other mutations had occurred.

Genomic DNA of the markerless mutant was isolated using the Wizard Genomic DNA Purification Kit (Promega, USA) with lysozyme (20 mg/ml), dissolved in water, and sequenced by the HiSeq 2500 platform (Illumina, USA).

*E. faecium* and its knockout mutant genomes were compared pairwise using Artemis Comparison Tool [58]. Pairwise comparison figures were drawn using EasyFig 2.2.2 [59]. GATK was used for variant calling to estimate the SNP differences between these two variants [71, 72].

### 2.11. GFP-Expression Plasmid in *Enterococcus*

A pEF25 vector with GFP cloned in the SmaI site resulting in pEF451 was transformed into *E. coli* EC1000, as described earlier (*under submission* [73]) and electroporated into competent *E. faecium* E1162 and the markerless isogenic mutant E1162Δ*tirE*. Transformants (GFP-E1162 and GFP-E1162Δ*tirE*) were selected under antibiotic pressure and confirmed by PCR as well as by a confocal laser scanning microscope (CLSM Leica SP5), equipped with an oil plan-Neofluar (×63/1.4 objective). GFP was excited at 395 nm.

### 2.12. Cell Lines and Culture

Peripheral blood mononuclear cells (PBMCs) (1 × 10^7^ cells/ml) and human neutrophils (PMN) (5 × 10^6^ cells/ml) were isolated from heparinized blood as described previously [65].

All mammalian cells were grown at 37°C in an incubator with 5% CO_2_. HEK293T cells, a human embryonic kidney cell line obtained from ATCC, USA, were used to stably express human TLR2, as described previously (*under submission* [74]). In short, human TLR2 (NM_003264.3) was cloned and its signal peptide (aa1-20) was replaced by the PreProTrypsin signal peptide (MSALLILALVGAAVA), adapted from the pFLAG-CMV-1 vector (Sigma-Aldrich, USA). To the N-terminus, a FLAG tag (DYKDDDDK) followed by a flexible linker (GGS) was attached. For stable expression in HEK293T cells, a lentiviral expression system was used. The FLAG tag was detected using anti-FLAG M2 antibody (Sigma-Aldrich, USA), subsequently stained with phycoerythrin-labeled goat-anti-mouse antibody, and detected on a flow cytometer (FACSVerse, BD Biosciences, USA). The HEK293T-TLR2 cells were cultured in Dulbecco's modified Eagle's medium (DMEM) with 10% (v/v) fetal calf serum (FCS) (Invitrogen Life Technologies, USA) and 100 units/ml of penicillin and streptomycin (Sigma-Aldrich, Germany).

Thp-1 cells (ATCC, USA), a human monocytic cell line, were maintained in the RPMI 1640 medium with 2 mM L-glutamine supplemented with 10% (v/v) fetal bovine serum (FBS; Biowest, USA), 10 mM HEPES, 1 mM sodium pyruvate, 4.5 g/L glucose, and 0.05 mM beta-mercaptoethanol (all from Gibco, Life Technologies, USA). For differentiation of Thp-1 to macrophage-like cells, RPMI 1640 with 2 mM L-glutamine without phenol red was used with the same supplements as above, but with addition of 25 nM phorbol 12-myristate 13-acetate (PMA).

### 2.13. Cytokine Production upon *E. faecium* Infection

The effect of the *tirE* locus on cytokine release was investigated by measuring IL-8 release upon Thp-1 infection with the *E. faecium* wildtype strain E1162 or E1162Δ*tirE*.

Bacteria were grown in BHI to OD_600_ 0.4, washed with PBS, and resuspended in the cell culture medium and diluted to the appropriate CFU/ml.

Thp-1 cells were seeded in a 24-well plate in RPMI at 1 × 10^5^ cells per well and differentiated for 24 h by addition of 25 nM PMA. For infection, 100 *µ*l of bacterial suspension (equal to MOI 300, MOI 100, and MOI 10) was added per cell culture well and incubated for 37°C. The supernatant was collected after 2, 4, and 6 h, and debris was spun down at 13000 rpm at 4°C.

IL-8 within the supernatant was detected using the IL-8 ELISA Kit (Thermo Fisher Scientific, USA) following the manufacturer's instructions. Absorbance was measured at 450 nm.

### 2.14. Interference with Ligand-Induced Cytokine Production

Mammalian cells were plated out at 2.5 × 10^4^ cells/well in a 96-well culture plate 24 h prior to the experiment to reach confluence. The inhibitor (TirEs and/or Hp1 or SSL3 [75]) was added at a final concentration of 10 *µ*g/ml, and the cells were incubated at 37°C for 1 h. After 6 h incubation with stimulus (3 ng/ml MALP-2 (Santa Cruz, USA); 3 ng/ml PAM-2-Cys and 3 ng/ml PAM-3-Cys (EMC Microcollections, Germany), the supernatant was collected and IL-8 was measured using a specific IL-8 ELISA Kit (Sanquin, the Netherlands) following the manufacturer's instructions. In short, Nunc MaxiSorp plates were coated with anti-IL-8, washed and blocked with 4% nonfat dry milk in PBS-T, and washed again and incubated with the supernatant in high-performance ELISA (HPE) buffer at 37°C for 1 h. After washing, biotinylated anti-IL-8 and subsequently streptavidin-HRP were added. Tetramethylbenzidine (TMB) was used as the substrate, and the reaction was stopped with sulfuric acid as soon as a change in color was visible. OD_450_ was measured for quantification.

### 2.15. Serum-Dependent Phagocytosis in Flow Cytometry

Blood was collected from healthy volunteers and allowed to clot. After centrifugation, the human serum was collected and pooled. The serum was stored at −70°C. When required, heat inactivation of serum was performed at 56°C for 30 min.

GFP-E1162 and GFP-E1162Δ*tirE* were grown in BHI to OD_600_ 0.4, washed with PBS, and exposed to serum for 15 min at 37°C in a 96-well round bottom plate (Greiner, Austria) on a shaking plateau prior to phagocytosis.

PMNs and PBMCs (200 *µ*l and 300 *µ*l, respectively) were added to the bacteria, and the cells were coincubated for 15 min at 37°C. The cells were thereafter fixed in 1.5% paraformaldehyde in RPMI-HSA, and fluorescence was measured through flow cytometry (FACSCalibur; Becton Dickinson, USA).

### 2.16. Intracellular Survival within Macrophages

Intracellular survival was assessed in a gentamicin-vancomycin protection assay. Thp-1 cells were seeded in a 12-well plate in RPMI with the required supplements at 1 × 10^6^ cells per well and differentiated for 24 h by addition of 25 nM PMA. Bacteria were grown in BHI to OD_600_ 0.4, washed with PBS, and resuspended in the cell culture medium. For infection, 200 *µ*l of bacterial suspension was added per cell culture well and incubated for 1 h. After PBS washing of Thp-1 cells, 16 *µ*g/ml vancomycin and 150 *µ*g/ml gentamicin were added to the wells in order to kill the extracellular bacteria. Thp-1 cells were thereafter harvested after 24, 48, 72, and 120 h and lysed in 0.3% saponin. The released intracellular bacteria were plated on blood agar plates and counted.

### 2.17. Whole-Blood Phagocytosis and Blood Survival Assay

GFP-E1162 and GFP-E1162Δ*tirE* from a blood agar plate were diluted in BHI to OD_600_ 0.1 and grown at 37°C to OD_600_ 0.4, washed with PBS, and resuspended in RPMI-HSA. Blood was obtained from a healthy volunteer in a hirudin blood tube (Roche Diagnostics, Switzerland) and diluted in RPMI-HSA. For all whole-blood experiments, blood was used at 80% if not otherwise indicated.

Phagocytosis was performed in round-bottom polystyrene tubes under rigorous shaking at 37°C for 20 min. Erythrocytes were lysed by adding FACS lysing solution (BD) for 20 min at 25°C. The remaining immune cells were washed with RPMI-HSA and fixed in 150 *µ*l 1% paraformaldehyde in RPMI-HSA. Fluorescence was measured through flow cytometry (FACSCalibur; Becton Dickinson, USA).

For blood survival assays, E1162 and E1162Δ*tirE* were grown in BHI to OD_600_ 0.4, washed with PBS, and incubated with 80% fresh hirudin blood in siliconized tubes (Sigma-Aldrich, USA) on a turning wheel at 37°C for 3 h, if not otherwise indicated. Blood cells were lysed in 0.3% saponin, and CFUs were counted on blood agar plates.

### 2.18. Statistical Analysis and Data Validation

Statistical data analysis was performed in GraphPad Prism 7. The presented data are expressed as mean ± SEM of pooled experiments. Blood experiments were performed at least in 3 different donors, representing both male and female.

### 2.19. Ethics Statement

The human blood analysis was carried out in accordance with ethical principles of the Helsinki Declaration, the Medical Ethics Committee of the University Medical Center Utrecht (the Netherlands), and ethical approval of (2014/1653) REK North-Norway with written informed consent of participants.

## 3. Results

### 3.1. Identification and Predicted Tertiary Structure of TirEs

Two putative proteins annotated with a TIR domain were identified in *E. faecium* in the protein database at NCBI. These two open reading frames (ORFs) were found, with a hypothetical protein (*hp1*) in between them. In *E. faecium* E1162, the TIR-domain-containing locus tags EFME1162_RS19585 and EfmE1162_RS19595 span 492 and 864 bp, respectively. In line with previous studies [19], the protein-encoding genes were designated *tirE1* and *tirE2*. The predicted mass of TirE1 was 18.5 kDa, TirE2 32.9 kDa, and Hp1 55.0 kDa (Table 1). The TIR-domains were verified through the CDD search [43] and found to belong to the protein families pfam08937 (TirE1) and pfam13676 (TirE2).

**Figure 1:**
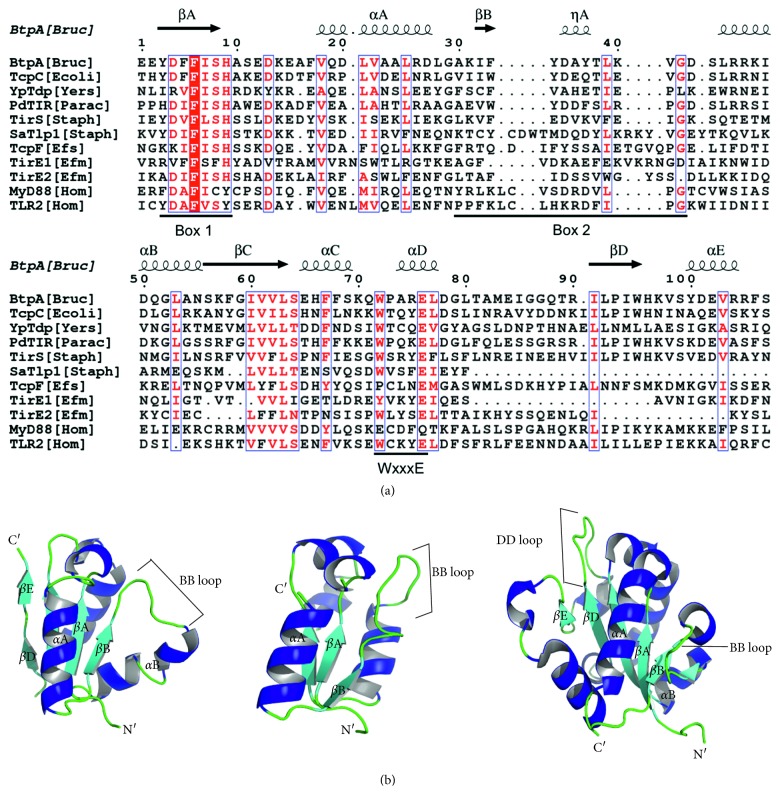
Secondary and tertiary structure prediction of TirEs. (a) Primary amino acid sequence alignment of TirE1 and TirE2 to bacterial TIR-domain-containing proteins and human TIR-domain-containing proteins. The region shown displays the TIR domain as predicted by CDD. Similar and identical residues are depicted in red and boxed in blue. Conservation in regions such as Box1, Box2, and the WxxxE motif is marked. The corresponding secondary structure elements of BtpA as determined by X-ray crystallography are shown on top of the alignment. (b) Predicted tertiary structures of TirE1 and TirE2 compared to the crystal structure of the TIR domain of MyD88. Key secondary structures are labeled; *α*-helices are shown in blue, *β*-sheets in cyan, and loops in green. The BB loop as a divergent structure is marked.

Amino acid sequence alignment of multiple bacterial TIR-domain-containing proteins showed high conservation of the TIR domain across species. Amino acid sequence was particularly conserved in boxes 1 and 2, as well as in the WxxxE motif. In TirE1, the WxxxE motif has a tyrosine instead of tryptophan, but both amino acids are aromatic. The WxxxE motif has been found in most bacterial TIR-domain-containing proteins including PdTir, SaTlp1, and TlpA, as well as eukaryotic TLR proteins TLR1, 2, 4, 6, and 10 and TIR adaptor protein SARM [76] (Figure 1(a)). Variability was observed in the region between strand *β*B and helix *α*B, the so-called BB loop (Figure 1(a)). The indicated BB loop has been described to be surface-exposed and essential for homotypic TIR-domain interactions in TLR/IL-1R signaling [77]. The predicted tertiary structure of both TirEs (Figure 1(b)) shows a typical flavodoxin-like TIR fold, with central parallel *β*-sheets surrounded by *α*-helices on both sides of the sheet [77].

A phylogenetic tree illustrating the relatedness of the aligned TIR-domain-containing proteins to each other showed that the human TLR2 and MyD88 are grouped in the same cluster, whereas the bacterial TIR-domain-containing proteins group in different clusters (Figure S2). Even though both TirE1 and TirE2 originate from *E. faecium*, they fall into two different clusters, indicating that these are probably not just gene duplications but two different genes.

### 3.2. The *tirE* Locus Is Prevalent in Nosocomial but Absent in Community Isolates

**Figure 2:**
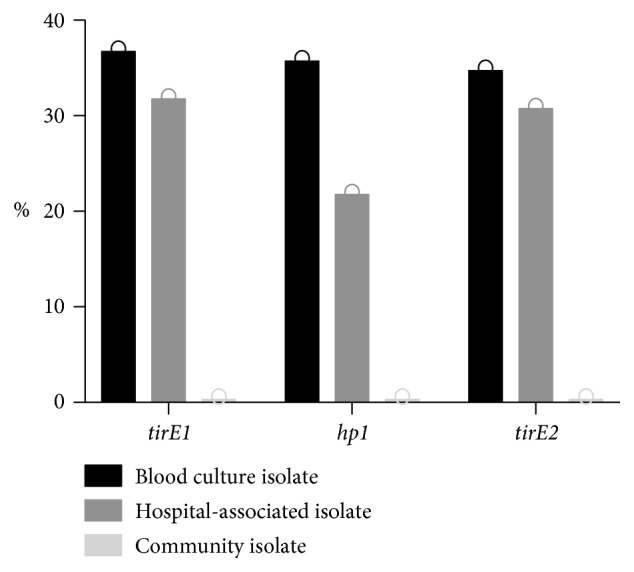
Prevalence of the genes *tirE1*, *hp1*, and *tirE2* in human *E. faecium* isolates. Percentage indicates the amount of isolates positive for *tirE1*, *hp1*, and *tirE2* among blood culture isolates (*n* = 161), other hospital-associated isolates (*n* = 875), and community isolates (*n* = 158).

A total of 1194 *E. faecium* isolates were screened for *tirE1*, *tirE2*, and *hp1* either by regular PCR using prevalence screening primers or by BLAST searches in sequenced isolates. *tirE1*, *hp1*, and *tirE2* genes were found to be highly prevalent in blood culture isolates (*tirE1* 37%, *hp1* 36%, and *tirE2* 35%), and among those, the dominant STs were ST17, ST203, and ST192. The genes were also frequent in other hospital-associated samples (*tirE1* 32%, *hp1* 22%, and *tirE2* 31%) with the most prevalent STs being ST78, ST389, and ST192. 84 of the hospital screening samples were positive for *tirE1* and *tirE2*, but lacked *hp1*, which coincides with the presence of *vanB* vancomycin-resistant cluster in 79 of them (Table S1). Contrasting the high prevalence in blood culture isolates and other hospital-associated samples, the three genes were absent in community isolates (Figure 2).

Furthermore, BLAST search revealed that the *tirE* locus was found in 14% of 460 draft genome sequences of nosocomial strains, while these genes were absent in draft genome sequences of all 56 community strains (Figure S3).

This dichotomy in the presence of the *tirE* locus between hospital and community isolates suggests that the possession of the *tirE* locus is beneficial for the bacterium in the hospital setting.

### 3.3. The *tirE* Locus Is Localized on a Putative Mobile Genetic Element of Phage Origin

**Figure 3:**
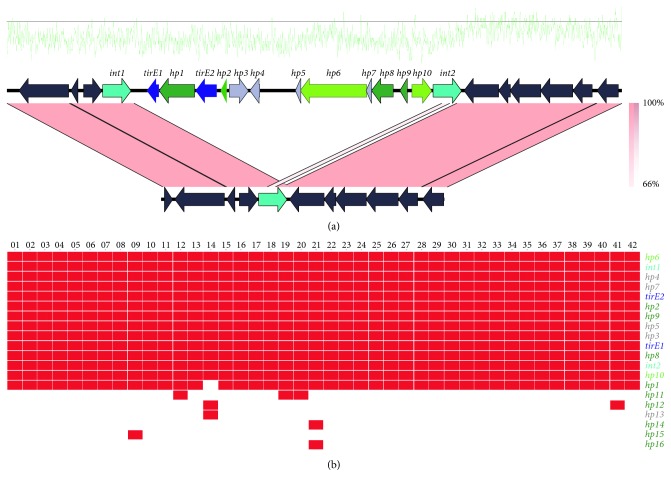
Comparative genomics of MGE-TirE insertion and relative abundance of MGE genes. (a) Pairwise alignment of E1162, containing MGE-TirE (top), and 17OM39. MGE-TirE is flanked by two integrases (cyan) and contains in total 14 open reading frames, color-coded by putative function. *tirEs* are shown in blue, putative DNA-binding hypothetical proteins in dark green, and putative transcription regulators in light green, hypothetical proteins (hp) for which no function could be predicted in grey. On top of the alignment, the GC abundance is illustrated in green. The black line refers to 50% GC. The percentage of nucleotide identity in forward and reverse strands is represented in light orange and dark orange, respectively, as shown in the bottom right of the alignment. (b) Gene presence/absence within MGE-TirE in 42 strains (top). Genes are color coded by the putative function of the proteins they encode (the same color code as in (a)).

Analysis of the genetic surroundings of the *tirE* locus suggested that the genes were localized on a putative mobile genetic element flanked by two integrases. This putative MGE was designated MGE-TirE (for mobile genetic element containing *tirE*s). Pairwise comparison of the genomic region encompassing MGE-TirE in the strain E1162 with the community strain *E. faecium* 17OM39 which does not contain the *tirE* locus revealed the MGE-TirE's size of 14.425 kbp and site of integration (Figure 3(a)). The putative function of the ORFs (*n* = 13) within MGE-TirEs is indicated in Figure 3(a) and in Table 1. Six of the ORFs were predicted to encode either transcription regulators (*n* = 2) or DNA-binding proteins (*n* = 4). 31% the GC content of MGE-TirE is especially low (GC content calculated in 4 representative strains), since the average GC content for *E. faecium* is 38% [78]. The two ORFs flanking MGE-TirE were predicted to encode putative integrases. They both showed high similarity to phage integrases. Furthermore, one of the hypothetical proteins (hp9) was similar to a *Lactobacillus plantarum* prophage remnant LP4 protein [79]. BLAST analyses of the region as a whole did not show any similarities to the sequences in the NCBI nonredundant database or the Sequence Read Archive. The deviant GC value and putative function of ORFs indicated presence of foreign genes, possibly of phage origin.

MGE-TirE was probably acquired multiple times during *E. faecium* evolution, as it occurs in different branches of the core genome phylogenetic tree (Figure S3). Moreover, MGE-TirE was integrated at an identical site adjacent to the host phage integrase gene (*int1*) in all the TirE-positive genomes, indicating site-specific integration. To describe the conservation of the content of MGE-TirE, a heat map was created, illustrating that most of the genes identified as part of MGE-TirE were highly abundant within the individual elements of all MGE-TirE-containing strains, including the integrases and TIR-domain-containing proteins (Figure 3(b)).

### 3.4. Expression of Genes of the *tirE* Locus

**Figure 4:**
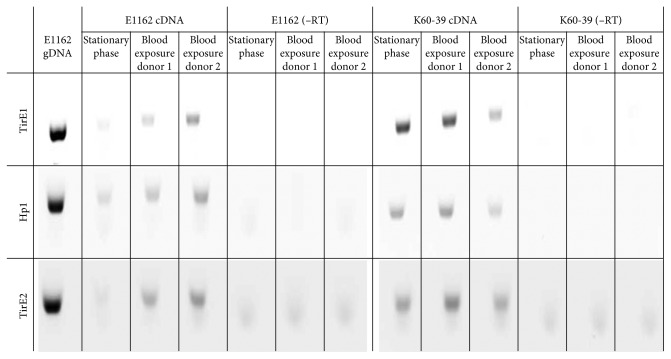
Expression of genes *tirE1*, *hp1*, and *tirE2* upon exposure of *E. faecium* to blood. RT-PCR products of E1162 and K60-39 grown to the stationary phase in BHI and upon 3 h exposure to human blood of two different donors are photographed. −RT shows the control of the PCR reaction without reverse transcriptase.

**Figure 5:**
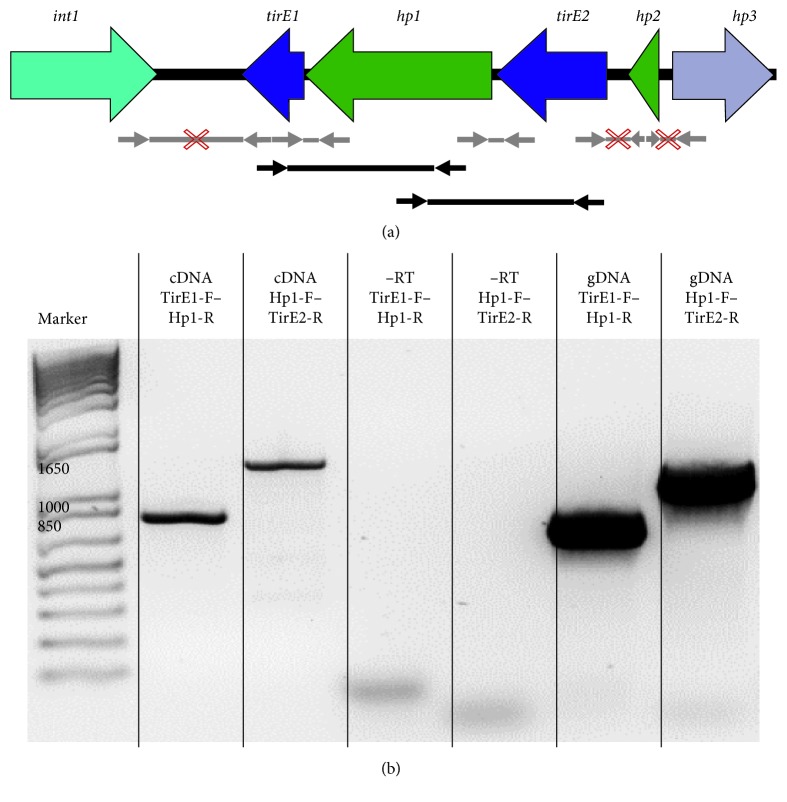
Both *tirE*s are part of one operon within E1162. (a) Graphical representation of the *tirE* operon within E1162 and primer locations. The ORFs in the immediate surrounding of *tirE*s are shown by thick arrows, while thin grey and black arrows indicate the location and orientation of the primers used for RT-PCR. Amplification is indicated as a straight line, and crossed lines indicate absence of products. Grey lines and arrows illustrate junction primers amplifying intergenic regions and their products. Black lines and arrows show the internal primers and their products, which were used to confirm linkage of the gene expression. (b) RT-PCR products linking *tirE1*, *hp1*, and *tirE2*. RT-PCR after amplification with *tirE1* internal (TirE1-F) plus *hp1* internal (Hp-R) primers yielded a 777 bp band and with *hp1* internal (Hp-F) plus *tirE2* internal (TirE2-R) gave a 1286 bp product. −RT shows corresponding negative controls and gDNA positive controls.

RT-PCR revealed that the genes of the *tirE* locus are expressed in both E1162 and K60-39. Expression was detected in the stationary phase in BHI and upon exposure to human blood for 3 h; all three genes showed enhanced expression in E1162 (Figure 4). Promoter prediction analysis revealed only weak promoters between the three genes of the *tirE* locus, so a linking RT-PCR, where the chosen primers hybridize to the different genes, was performed to determine whether the genes are part of a single operon (Figure 5(a)). The junctions of *tirE1* and *hp1* as well as *hp1* and *tirE2* gave products (Figure 5(b)), whereas the outward-pointing junctions did not (data not shown). Sequencing confirmed the identity of the PCR products (data not shown). This indicates that the two *tirE*s and *hp1* encoded by the same strand are transcribed together.

### 3.5. *tirE*-Locus-Encoded Proteins Are Released into Supernatant and Associated with Membrane Vesicles

**Figure 6:**
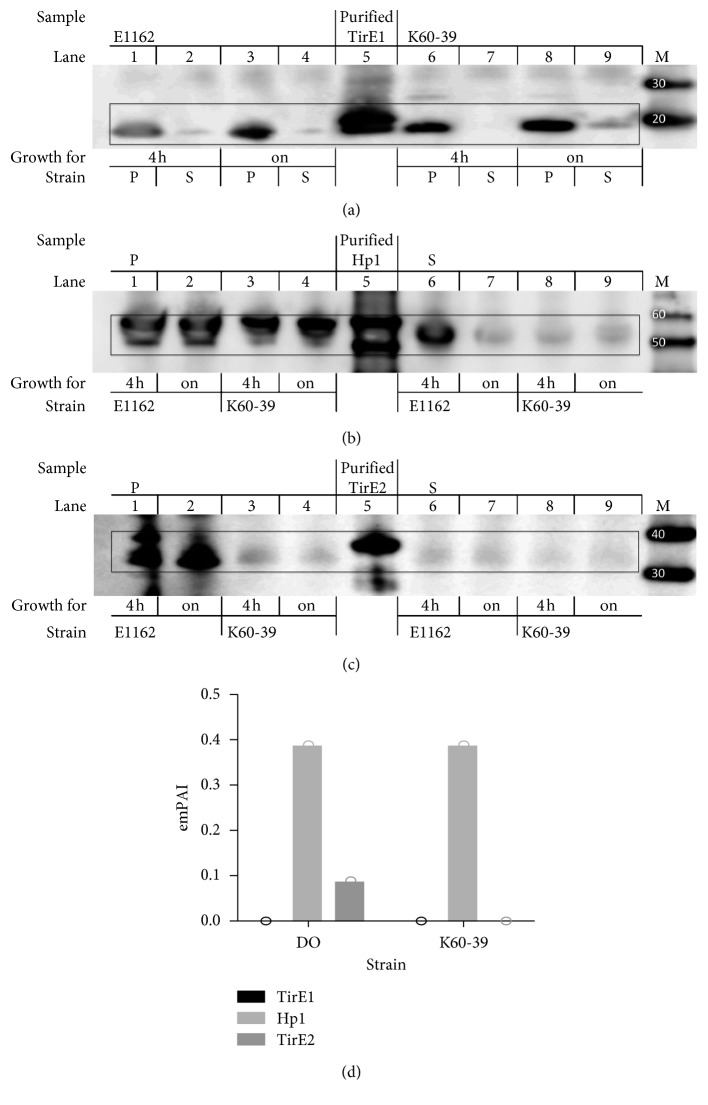
Detection of TirEs and Hp1 in the bacterial cell lysate, supernatant, and MV. (a–c) TirE1 (18.5 kDa), Hp1 (55 kDa), and TirE2 (33 kDa) were immunoprecipitated from the lysed pellet (P) or bacterial supernatant (S) after 4 h or from overnight culture (on) and detected in western blot with anti-TirE1, anti-Hp1, or anti-TirE2 serum. Shown are samples from the E1162 and K60-39, purified proteins (TirE1 with his tag 20.4 kDa (lane 5 in (a)), Hp1 with his tag 57 kDa (lane 5 in (b)), and TirE2 with his tag 35 kDa (lane 5 in (c))), and Magic marker (M) with size indicated in kDa. (d) EmPAI values indicating the protein abundance of TirE1, Hp1, and TirE2 proteins in membrane vesicles derived from K60-39 and DO.

Since there were no signal peptide cleavage sites predicted by SignalP in TirEs and Hp1, it was an open question whether bacteria were able to release these proteins (Table 1). Antibodies against TirEs and Hp1 were specific but unfortunately had low sensitivity (results not shown). In order to detect protein release, anti-TirE/Hp1 serum was used to immunoprecipitate the proteins from the bacterial lysate and supernatant. The immunoprecipitated proteins were thereafter detected by immunoblot. All three proteins were detected in the cell lysate as well as in the supernatant of both E1162 and K60-39 cultures, indicating release through an unknown mechanism (Figures 6(a)–6(c)).

Membrane vesicles (MVs) have previously been suggested as a secretory system [80]. TirE2 and Hp1 were found to be associated with MVs from K60-39 and DO. The exponentially modified protein abundance index (emPAI) value for TirE2 was 0.389 and for HP1 was 0.089 (Figure 6(d)). The identification confidence was characterized as “high” in the protein false discovery rate (FDR) confidence, and the FDR was lower than 1% with 1 peptide and 2 peptide-to-spectrum matches [67].

### 3.6. Construction of a *tirE1-hp1-tirE2* Knockout Strain for Functional Studies

A markerless mutant lacking the *tirE* locus was constructed and confirmed by PCR, gene expression, and whole-genome sequencing (Figures S4A–C). Comparison of the whole-genome sequences of the wildtype strain (E1162) and mutant strain (E1162Δ*tirE*) confirmed the absence of *tirE1-hp1-tirE2* (Figure S4C), and except for these three genes, the strains share 99% nucleotide identity (data not shown). The deletion of *tirE1-hp1-tirE2* did not affect the bacterial growth in BHI (Figure S4D). Unfortunately, we did not succeed in constructing single gene knockouts nor in complementing the deletion, probably because there were repetitive sequences in this gene region and it has a very low GC content.

### 3.7. The *tirE* Locus Does Not Interfere with Cytokine Release

**Figure 7:**
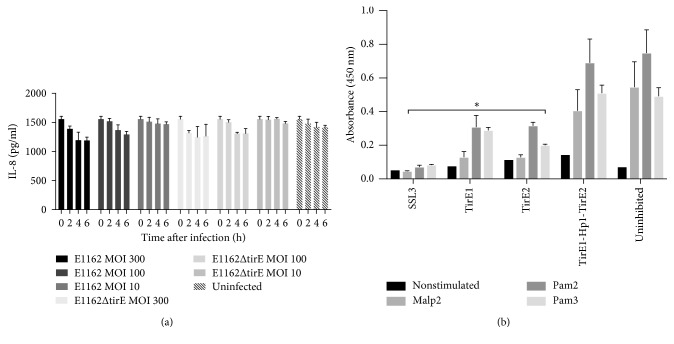
Effect of TirEs and Hp1 on IL-8 release. (a) Presence of *tirE1-hp1-tirE2* does not affect IL-8 release. Upon infection of macrophage-like cells (differentiated Thp1 cells) with E1162 and E1162Δtir at MOI 300, MOI 100, and MOI 10, IL-8 was measured in the cellular supernatant at 2 h, 4 h, and 6 h after infection, through IL-8 ELISA. Data are pooled from 3 independent experiments, each in duplicates. (b) Interference of TirE1 and TirE2 with TLR2 signaling. IL-8 release was measured as a response of HEK293 cells stably expressing TLR2 towards stimulation with the lipoproteins Malp2, Pam2Cys, and Pam3Cys. The cells were left untreated (nonstimulated) or were added Malp2, Pam2Cys, or Pam3Cys. One hour before stimulation with agonists, the TLR2-expressing HEK293 cells were added SSL3 (well-known inhibitor of TLR2 signaling), TirE1, TirE2, or a combination of TirE1, Hp1, and TirE2 to a final concentration of 10 *µ*g/ml. IL-8 was quantified in IL-8 ELISA, using TMB as the substrate and measuring the absorbance at 450 nm. Data are shown in triplicates, representative of 3 independent experiments. ^*∗*^ *P* ≤ 0.05, inhibited vs uninhibited: *P*_Tir1Malp2_=0.051, *P*_Tir1Pam2_=0.045, *P*_Tir1Pam3_=0.017, *P*_Tir2Malp2_=0.049, *P*_Tir2Pam2_=0.035, and *P*_Tir2Pam3_=0.004; Abs: absorption.

Differentiated Thp1 cells were infected with E1162 or its isogenic mutant E1162Δ*tirE* at MOI 10, 100, and 300. The IL-8 was then detected in the supernatant. As seen in Figure 7(a), E1162 and E1162Δ*tirE* at all MOIs induced IL-8 release in a similar way.

To examine whether TirEs are capable of interfering with TLR2 signaling, HEK293T cells stably expressing TLR2 were stimulated with the synthetic lipopeptides MALP-2, Pam2Cys, and Pam3Cys in the presence or absence of the recombinant TirEs and Hp1, which was added to the cells before stimulation. IL-8 was measured in the supernatant through ELISA. TirE1 and TirE2 both inhibited Malp2-, Pam2Cys-, and Pam3Cys-induced TLR2 activation significantly (Figure 7(b)). This effect was similar to the inhibition by SSL3, a well-known staphylococcal inhibitor of TLR2 signaling [75]. However, the combination of TirE1, Hp1, and TirE2 had no inhibitory effect on TLR2-mediated release of IL-8 (Figure 7(b)).

### 3.8. The *tirE* Locus Does Not Have an Effect on Phagocytosis or Bacterial Survival within Macrophages

Since phagocytosis of enterococci is serum dependent [81–83], the effect of the possession of the *tirE* locus on serum-induced phagocytosis was further examined. Hence, the GFP-expressing enterococci (GFP-E1162 and GFP-E1162Δ*tirE*) were incubated for 1 h in human blood, before the level of fluorescent bacteria within PMNs was analyzed by flow cytometry. There was no significant difference in the level of phagocytosis of the wildtype strain and its isogenic mutant (Figure S5A). The experiment was then repeated using blood cells. Here, GFP-E1162 and GFP-E1162Δ*tirE* were opsonized by adding 50% or 100% serum before being exposed to human PMNs and PBMCs. Again, there was no significant difference between the phagocytosis of GFP-E1162 and GFP-E1162Δ*tirE* (Figure S5B). This clearly shows that the *tirE* locus does not influence the host-mediated phagocytosis of the bacteria.

Next, the role of the *tirE* locus for intracellular survival in macrophages was investigated. E1162 and E1162Δ*tirE* enterococci were coincubated with Thp-1 over time, before being plated out for CFU determination. 0.5% of E1162 and E1162*ΔtirE* survived after 24 h, and after 120 h, very few bacteria had survived. The presence or absence of the *tirE* locus did not have any effect on the ability of the bacteria to survive in macrophages (Figure S6).

### 3.9. Presence of the *tirE* Locus Enhances Bacterial Survival and Proliferation in Blood

**Figure 8:**
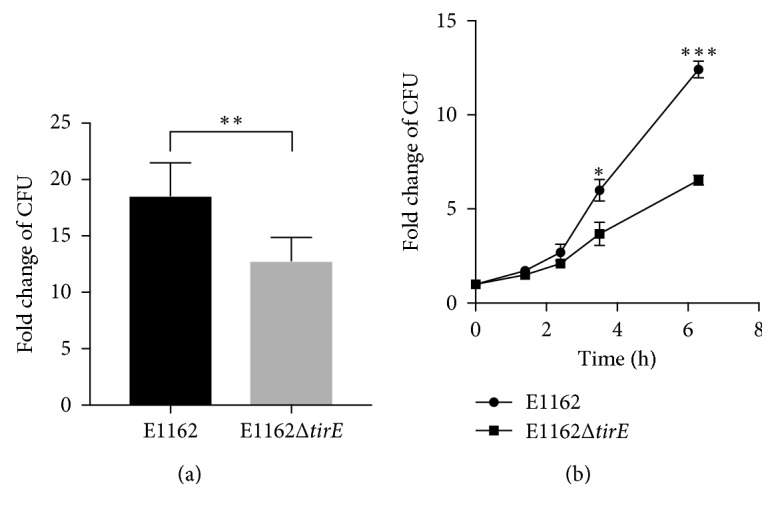
Presence of *tirE1-hp1-tirE2* increases bacterial survival and proliferation in human whole blood *ex vivo*. (a) *tirE1-hp1-tirE2* enhances bacterial proliferation in human whole blood. After 3 h of exposure of E1162 and E1162Δ*tirE* to whole blood, the fold change in CFU is significantly higher for E1162 compared to E1162Δ*tirE*, where the inoculum is set as 1. Data shown represent pooled data from 3 donors (inoculum 20 *µ*l at OD_600_ 0.4, *t*-test, 2-tailed, *P*_3*h*_=0.002). (b) *tirE1-hp1-tirE2* enhances proliferation in human whole blood over time. E1162 and E1162Δ*tirE* were incubated in blood for indicated time points. Data represent quadruplicates from one healthy donor and are representative of 3 independent experiments in different donors. (inoculum = 20 *µ*l at OD_600_ 0.4, *t*-test, *P*_3.5*h*_=0.0328, *P*_6.3*h*_ < 0.0001).

Finally, it was evaluated whether the presence of the *tirE* locus plays a role in *E. faecium*'s survival in blood. E1162 and E1162Δ*tirE* were incubated in human whole blood for 3 h before plating for CFU determination. The wildtype strain showed a 19-fold increase in CFU over the blood incubation, while its isogenic mutants showed only a 13-fold increase in CFU (Figure 8(a)). Additionally, the differences in survival and replication between E1162 and E1162*ΔtirE* increased over time (up to 6 h) (Figure 8(b)). This suggests that the presence of the *tirE* locus is beneficial for bacterial growth in human blood.

## 4. Discussion

The pronounced increase in bacteremia caused by *E. faecium* [7, 84] and the limited knowledge about enterococcal virulence factors led to the search for TIR-domain-containing proteins in this species. This study identified a *tirE* locus encoding two TirE proteins and an intermediate protein Hp1. The *tirE* locus was prevalent in nosocomial isolates but absent in commensals (Figure 2) and located on a putative mobile genetic element of phage origin (Figure 3). The three genes may be expressed as an operon (Figure 5), and presence of all three proteins was confirmed in the bacterial cell lysate and supernatant (Figure 6). Moreover, the *tirE*-locus genes were expressed when bacteria were exposed to blood (Figure 4), and the presence of the *tirE* locus increased bacterial proliferation in blood (Figure 8).

Hp1 intersected the two genes encoding the two TirE proteins presented here. Since we were able to link two of these genes by RT-PCR and no strong promoter was identified between the genes, we suggest that they are expressed as one operon. The *tirE* locus was found on a putative mobile genetic element. The element is putatively of phage origin because of its low GC content and the presence of phage proteins. Likewise, *tcpF* of *E. faecalis* is found in a hotspot of recombination events of phage origin [24]. In fact, it has been observed multiple times that bacterial *tir* genes are found in regions of phage origin, e.g., *tlpA* (*S. enterica*) [18], *tcpC* (*E. coli*) and *tcpB* (*Brucella*) [85], or on mobile elements such as *tirS* (*S. aureus*) on SCC [86]. Thus, lateral transfer of bacterial *tir* genes is likely. Furthermore, the putative MGE-TirE appeared in multiple branches within the *E. faecium* phylogenetic tree (Figure S3), indicating horizontal gene transfer. However, it cannot be concluded whether the element was acquired or lost several times.

A high prevalence of the *tirE* locus was found in clinical and blood culture isolates, whereas the genes are absent in community isolates (Figure 2). Similar observations were made for *tcpC*, which was present in 40% of uropathogenic strains versus 8% in commensal strains [85]. *TcpF* is predominant among urinary tract infection isolates but also frequent in commensal isolates (76% in invasive versus 58% in commensal strains) [21]. This could however be explained by the differences in the clade structure of *E. faecium* and *E. faecalis*: while *E. faecium* is characterized by host-specific lineages and clades, with a clearly distinct clade (A1) of isolates originating from hospitalized patients, this divergence is not seen in *E. faecalis* [1, 87]. In this context, it makes sense that *E. faecium tirE* is not found in community isolates, while *E. faecalis tcpF* is. For *E. faecalis*, it was suggested that the possession of the *tcpF* gene is also beneficial in community isolates, since the homeostasis in the gastrointestinal tract is crucial for commensals. Accordingly, avoiding NFkB activation through TLR signaling inhibition reduces the risk of homeostatic imbalance [21]. Besides the high prevalence of the *tirE*-locus genes in clinical and blood culture, it was observed that 10% of the hospital screening samples positive for *tirE1* and *tirE2*, but lacking *hp1*, were also positive for the *vanB* vancomycin-resistant cluster (Table S2). *hp1*, alias HMPREF0351_10592, was previously described as an insertion site for the conjugative *vanB* transposon Tn*1549*/*5382* in different strain backgrounds (ST192, ST78, ST927, and ST202 isolates) [88]. This *vanB* transposon has also been shown to prefer AT-rich sequences [89], and the putative MGE-TirE with its 31% GC content is AT enriched. However, it remains to be investigated by future studies whether *hp1* provided an insertion site for the *vanB* transposon in our strain collection.

No direct bacterial contact with the host cell is needed to exert the function of bacterial TIR-domain-containing proteins, e.g., for *E. coli* TcpC [90] and *S. aureus* TirS [19], and bacterial TIR-domain-containing proteins have been detected inside host cells previously [85]. However, most of the bacterial TIR-domain-containing proteins lack secretion signals [22], and how the proteins are released and transferred to other cells remains largely unknown. Here, we detected all *tirE*-locus-encoded proteins in the bacterial supernatant and demonstrated that TirE2 and Hp1 can be found associated with bacterial MVs. Similarly, *S. aureus* TirS was also associated with MVs isolated from bacteria grown in BHI (results not shown). This might suggest that bacterial TIR-domain-containing proteins can be released through MVs. In order to test this hypothesis, the vesicular proteomic contents of other *tir*-gene-possessing bacteria should be evaluated and compared to the nonmembrane bound secretome.

This study found that purified TirEs negatively interfere with IL-8 release of HEK293T cells expressing TLR2 when added to the cell cultures prior to stimulation with its specific ligands (Figure 7(b)). These findings are in line with results from NFkB-reporter assays for *E. faecalis* TcpF, conducted in hTLR2-expressing HEK293 cells stimulated with PamCys3 [21], and with similar studies on *S. aureus* TirS [19, 86]. Bacterial TIR-domain-containing proteins from Gram-negative bacteria [18, 22, 90] have also been shown to impede the host innate immune response through interference with TLR signaling. The inhibitory effect of TirEs on IL-8 release was abolished when a combination of TirEs and Hp1 was used. Similarly, there was no difference in cytokine release in macrophage-like cells infected with E1162 or E1162*ΔtirE* (Figure 7(a)). The mechanism behind this phenomenon is elusive but might involve a regulatory role of Hp1. Unfortunately, it was not possible to make single knockouts to distinguish between effects of the proteins. Two staphylococcal TIR-domain-containing proteins, SaTlp1 and SaTlp2, found in zoonotic ST398, also showed a deviation from the typical theme: they upregulated NFkB signaling instead of downregulating it [20].

In *E. faecium*, the *tirE* locus promoted bacterial proliferation in human blood (Figure 8) and blood also induced *tirE* expression (Figure 4). This suggests that the *tirE* locus provides *E. faecium* with an advantage of surviving in blood by interfering with TLR-2 signaling. This could also explain the predominance of *tirE* and *hp1* in blood culture and hospital isolates.

## 5. Conclusion

The presented study describes TirEs, *E. faecium* TIR-domain-containing proteins, as novel virulence factors. The *tirE* locus is exclusive of *E. faecium* nosocomial isolates and located on a putative mobile genetic element of phage origin. Bacterial proliferation within human blood is enhanced by possession of the *tirE* locus, implicating a role of TirEs in bacteremia and the pathogenicity of *E. faecium*.

## Figures and Tables

**Table 1:** ORFs in the putative MGE-TirE of *E. faecium* strain E1162.

Locus tag in E1162	Product NCBI	CDD (Conserved Domain Database)	Phyre2 prediction (putative function)	SignalP prediction (secretion signal)	Size (Da)	Name
EFME1162_RS19580	Site-specific integrase	DNA-binding integrase	Integrase	—	44551	int1
EFME1162_RS19585	—	TIR-like	TIR domain	—	18496	tirE1
EFME1162_RS19590	—	Sir2-like	Nucleotide binding	—	55003	hp1
EFME1162_RS19595	—	TIR-like	TIR domain	—	32901	tirE2
EFME1162_RS19600	Transcription regulator	DNA binding	DNA binding	—	9466	hp2
EFME1162_RS19605	—	—	—	Yes	29307	hp3
EFME1162_RS0109495	—	—	—	—	12407	hp4
EFME1162_RS19610	—	—	—	—	8588	hp5
EFME1162_RS19615	—	DnaJ super family	Transcription regulator	Yes	98796	hp6
EFME1162_RS0109500	—	—	—	—	8445	hp7
EFME1162_RS19620	—	—	DNA binding	—	34840	hp8
EFME1162_RS19625	DNA binding protein	DNA binding	DNA binding	—	11591	hp9
EFME1162_RS19630	Transcription regulator	Transcription regulator	Transcription regulator	—	31516	hp10
EFME1162_RS19635	Site-specific integrase	Integrase	Integrase	—	44970	int2

## Data Availability

The datasets analyzed during the current study are included in the supplementary material file and are available from the corresponding author upon reasonable request.

## Supplementary Materials

Supplementary Materials Figure S1: expression and purification of TirE1 and TirE2. Figure S2: phylogenetic tree of eukaryotic and prokaryotic TIR-domain-containing proteins. Figure S3: midpoint rooted tree displaying the presence and absence of *tirE1-hp1-tirE2*. Figure S4: confirmation of successful construction of E1162ΔtirE. Figure S5: *tirE1-hp1-tirE2* does not influence the immune cells ability to phagocytose the bacteria. Figure S6: presence of *tirE1-hp1-tirE2* does not influence bacterial survival within macrophages. Table S1: prevalence of the *tirE* locus. Table S2: strains and plasmids used for laboratory experiments. Table S3: primers used in the study. Table S4: metadata of *E. faecium* genomes harboring *tirE1-hp1-tirE2* genes.
