# Supplementary material for: Enterococcus faecium TIR-Domain Genes Are Part of a Gene Cluster Which Promotes Bacterial Survival in Blood
Source: Int J Microbiol. 2018 Dec 3;2018:1435820. doi: 10.1155/2018/1435820 (PMC6304867; doi:10.1155/2018/1435820)
Supplement: Supplementary Materials — Figure S1: expression and purification of TirE1 and TirE2. Figure S2: phylogenetic tree of eukaryotic and prokaryotic TIR-domain-containing proteins. Figure S3: midpoint rooted tree displaying the presence and absence of tirE1-hp1-tirE2. Figure S4: confirmation of successful construction of E1162ΔtirE. Figure S5: tirE1-hp1-tirE2 does not influence the immune cells ability to phagocytose the bacteria. Figure S6: presence of tirE1-hp1-tirE2 does not influence bacterial survival within macrophages. Table S1: prevalence of the tirE locus. Table S2: strains and plasmids used for laboratory experiments. Table S3: primers used in the study. Table S4: metadata of E. faecium genomes harboring tirE1-hp1-tirE2 genes. [file 1435820.f1.pdf]

Supplementary Figures

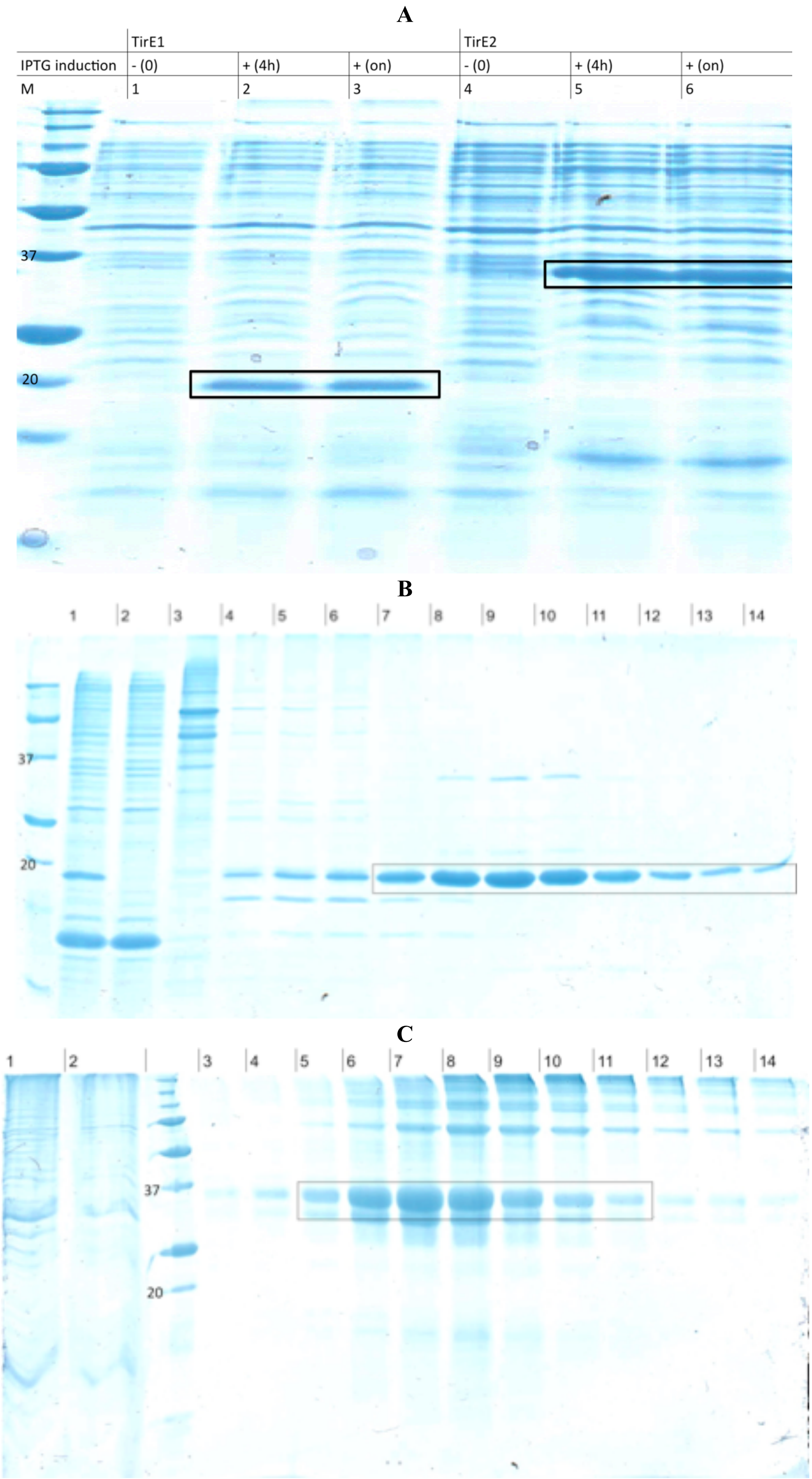

Figure S1: Expression and purification of TirE1 and TirE2. (A) *E. coli* Rosetta Gami containing expression plasmid encoding TirE1 or TirE1 were left untreated or induced by IPTG for 4 hours or overnight. Aliquots of bacterial lysates were run on SDS PAGE. TirE1 and TirE2 are indicated in boxes. (B) SDS PAGE gel showing aliquots of bacterial lysate after induction of TirE1 expression with IPTG (lane 1), the flow through (lane 2) and the fractions of native purification of TirE1 on a nickel column (lane 3-14). The TirE1 protein containing fractions (marked by a box) were pooled and re-buffered to PBS through dialysis. (C) SDS PAGE of aliquots after denaturizing purification of TirE2. The gel and samples were handled as indicated in B.

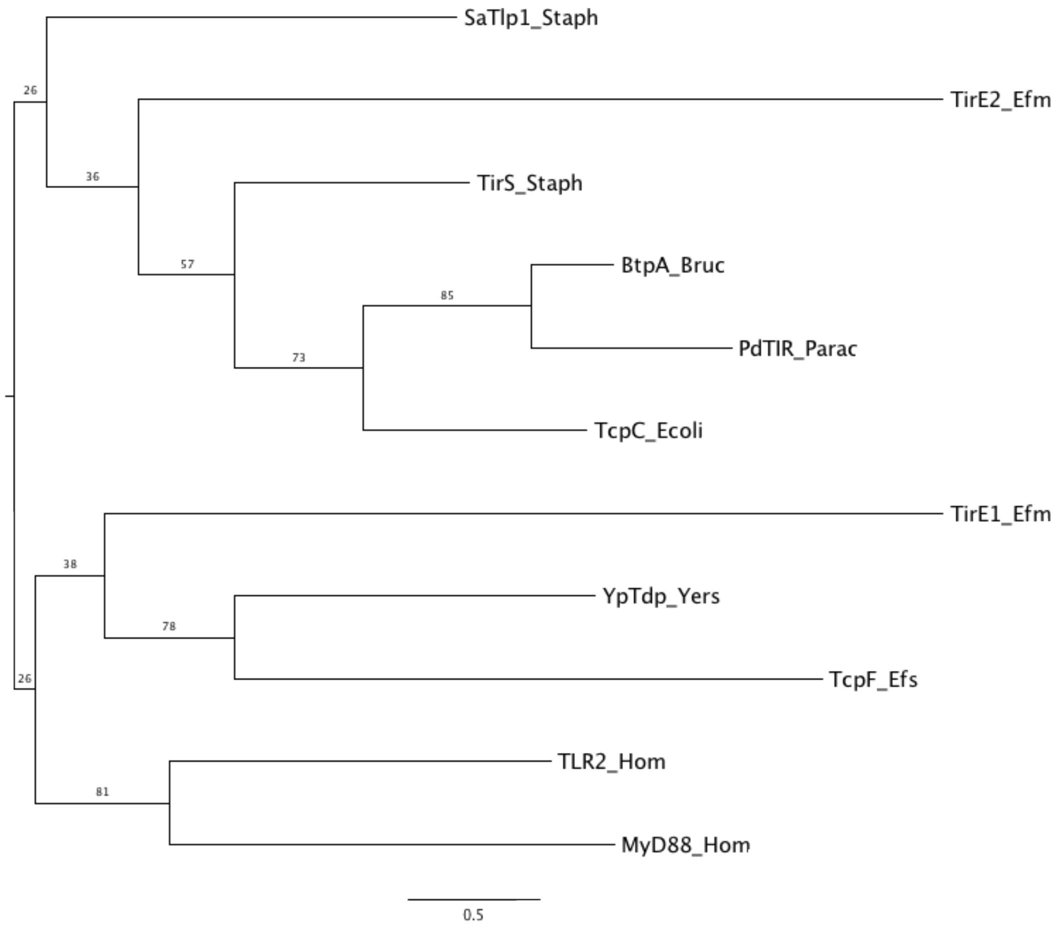

Figure S2: Phylogenetic tree of eukaryotic and prokaryotic TIR-domain containing proteins. Midpoint-rooted maximum-likelihood phylogram with 100 bootstraps based in TIR-domain containing proteins. TLR2 and MyD88 are human proteins while the rest are of bacterial origin.

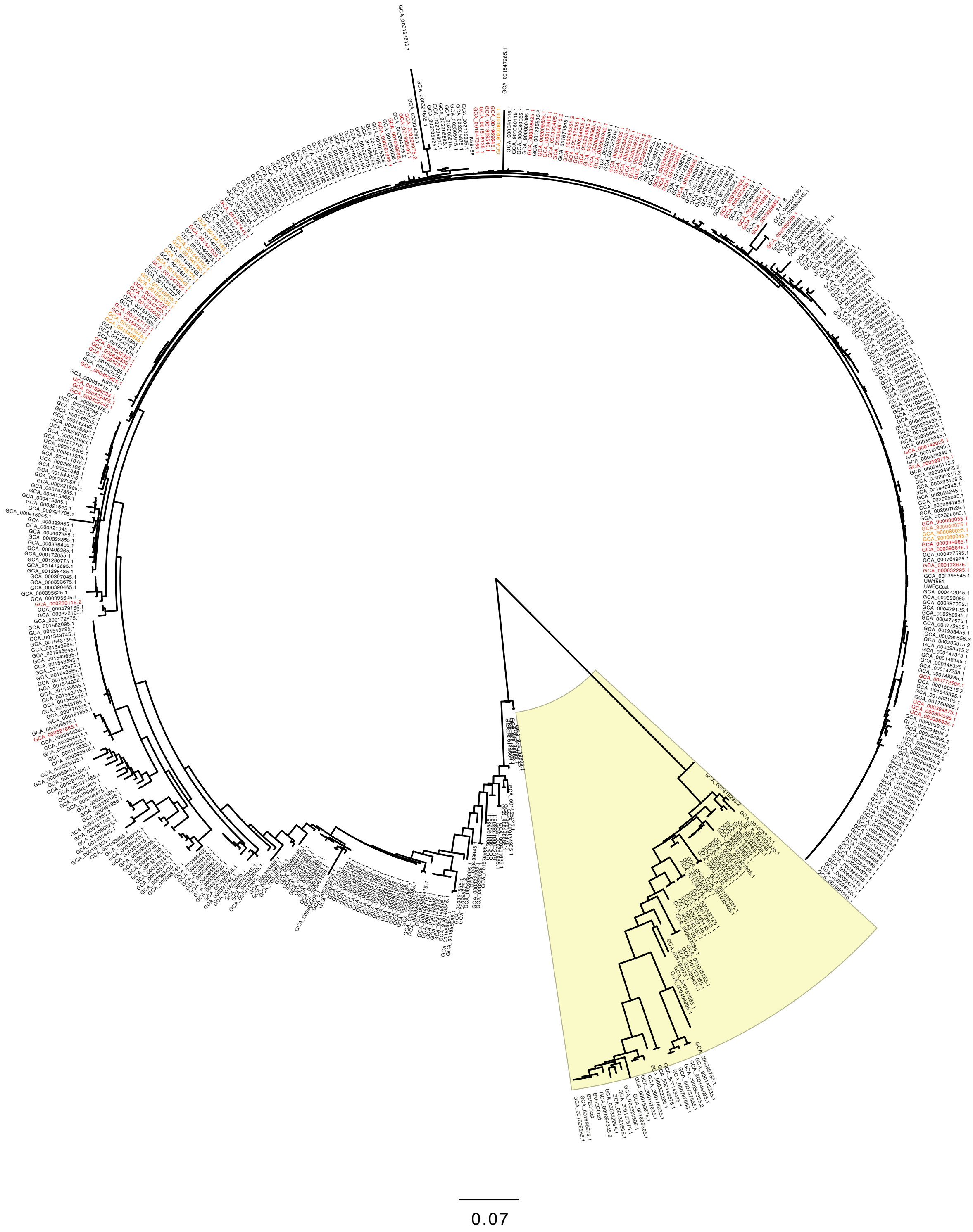

Figure S3: Mid-point rooted tree displaying the presence and absence of *tirE1-hp1-tirE2*. Presence of all three genes is shown in red, partial presence of only one or two genes is shown in orange and the absence of *tirE1-hp1-tirE2* is shown in black. Community strains are highlighted in a yellow box. (Nosocomial strains n = 460, community strains n = 56)

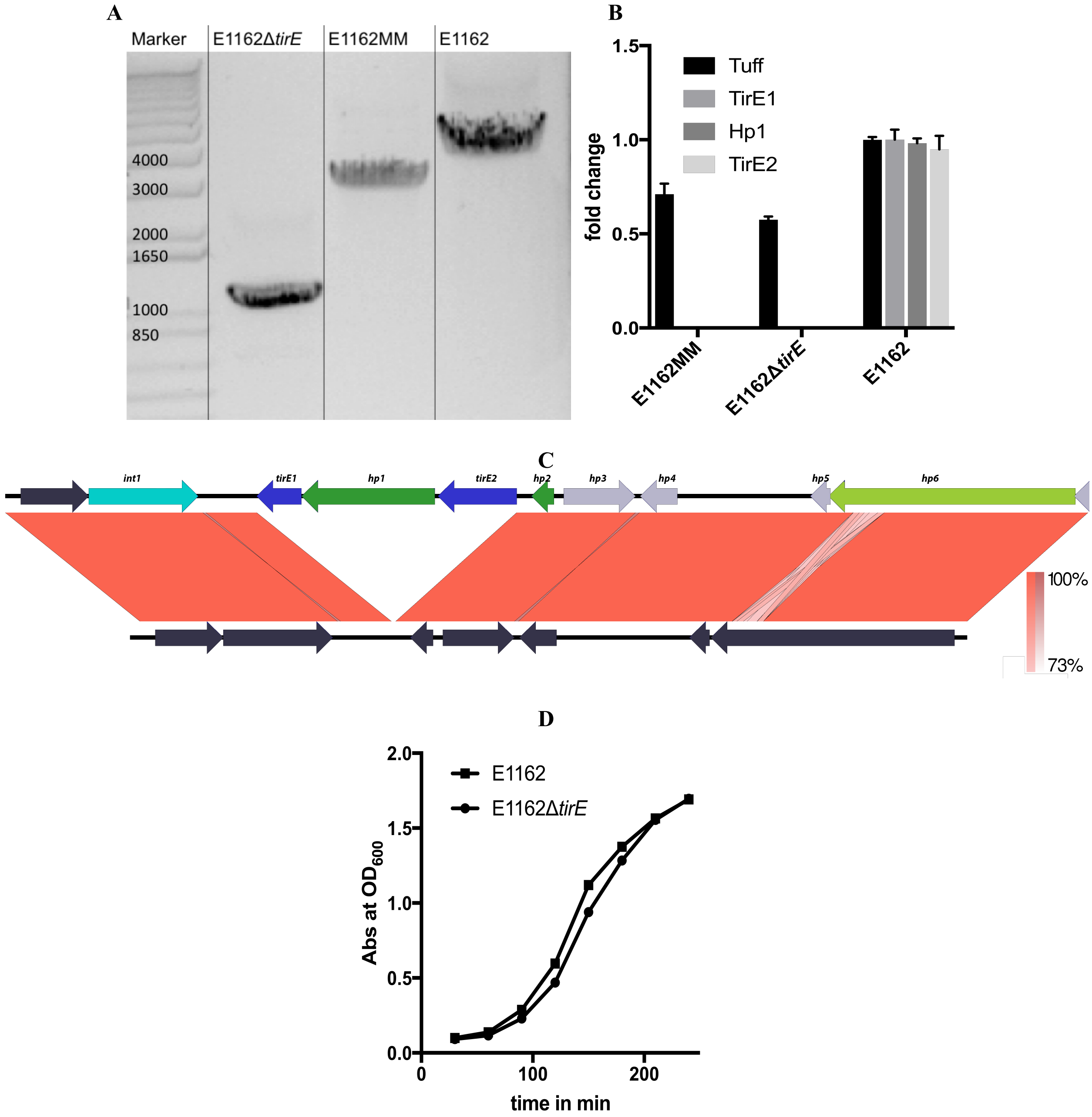

Figure S4: Confirmation of successful construction of E1162Δ*tirE*. (A) PCR of markerless-mutant construction products. PCR product of TirE region in E1162Δ*tirE* (*tir1-hp1-tir2* knocked out to obtain a markerless mutant), E1162MM (marked mutant, *tir1-hp1-tir2* is replaced by gentamicin resistance gene) and E1162 wild type. Products were amplified from genomic DNA using primers outside of the region (Tir1 check up, TirE2 check down, expected product size E1162Δ*tirE* 1 kb, E1162MM 3 kb and E1162 4 kb). (B) qPCR of knocked out genes in of E1162 and E1162Δ*tirE*. Fold change of expression of TirE genes in E1162, MM and E1162Δ*tirE* measured in qPCR on cDNA from RNA of the respective strain. Internal primers for the respective genes were used. *Tuff* was used as a positive control house keeping gene and reference to calculate the fold change of expression. (C) Alignment of *tirE* region of E1162 and E1162Δ*tirE*. WGS data illustrating the difference in *tirE* region between E1161 and E1162Δ*tirE*. Colors and ORFs are as introduced in Figure 3 (*tirE*s shown in dark blue). The percentage of nucleotide identity in forward and reverse strands is represented in light and dark orange respectively, as shown in the bottom right of the alignment. Contig comparisons and read mapping with subsequent single-nucleotide polymorphism (SNP) and variation calling showed no other genomic alterations between these isogenic isolates (data not shown). (D) Growth curve of E1162 and E1162Δ*tirE* in BHI (Abs – absorption).

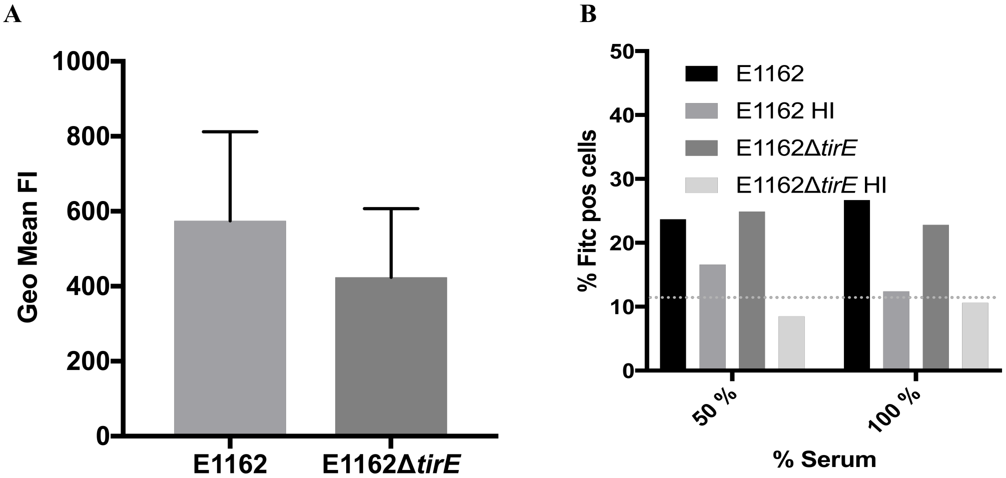

Figure S5: *tirE1-hp1-tirE2* does not influence the immune cells ability to phagocytose the bacteria. (A) Absence of *tirE1-hp1-tirE2* does not influence bacterial phagocytosis in whole blood. The geometric mean of fluorescence intensity is given for PMNs in whole blood from 3 donors in 80% blood. (FI – fluorescence intensity). (B) Absence of *tirE1-hp1-tirE2* does not influence PMN or PBMCs – mediated serum enhanced phagocytosis. GFP-expressing E1162 and E1162Δ*tirE* were pre-opsonized with serum before phagocytosis by PMNs and PMBCs (amount of phagocytosis without serum is indicated as a dashed line). Heat-inactivated (HI) serum-opsonized bacteria were included as control.

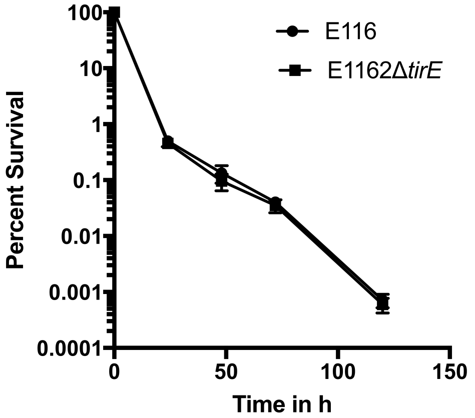

Figure S6: Presence of *tirE1-hp1-tirE2* does not influence bacterial survival within macrophages. E1162 and E1162Δ*tirE* were co-incubated with Thp1-derived macrophages for 24, 48, 72 and 120 h. The bacterial inoculum was arbitrary set as 100%, and the number of surviving bacteria calculated as the percentage of inoculum.

Supplementary Tables

Table S1: Prevalence of the *tirE* locus

	Name	Year	Country	Isolation Source	MLST	*tirE1*	*hp1*	*tirE2*	*van*
**Positive for all 3**	50694469	2013	NOR	Hospital screening sample	unknown	1	1	1	?
	E0155	1995	USA	Hospital screening sample	17	1	1	1	?
	E0470	1999	NLD	Hospital screening sample	16	1	1	1	?
	E1644	2002	DEU	Hospital screening sample	78	1	1	1	?
	TUH 51-65	2006	PRT	Hospital screening sample	78	1	1	1	?
	VRE-106	2007	DNK	Hospital screening sample	78	1	1	1	?
	VRE0673	2008	SWE	Hospital screening sample	78	1	1	1	?
	E6901	2010	CHE	Hospital screening sample	78	1	1	1	?
	E8416	2015	NLD	Hospital screening sample	412	1	1	1	*vanA*
	E1165	1997	ITA	Hospital screening sample	17	1	1	1	None
	E4853	2009	CHE	Hospital screening sample	78	1	1	1	?
	E6704	2010	CHE	Hospital screening sample	192	1	1	1	?
	E7208	2008	GRC	Hospital screening sample	203	1	1	1	?
	E7215	2009	GRC	Hospital screening sample	412	1	1	1	?
	V97	2013	DNK	Hospital screening sample	192	1	1	1	?
	E6045	2010	PRT	Hospital screening sample	78	1	1	1	?
	E5835	2009	NLD	Hospital screening sample	203	1	1	1	?
	E5834	2009	NLD	Hospital screening sample	203	1	1	1	?
	E5833	2009	NLD	Hospital screening sample	203	1	1	1	?
	E6047	2010	PRT	Hospital screening sample	78	1	1	1	?
	E5836	2009	NLD	Hospital screening sample	203	1	1	1	?
	E5837	2009	NLD	Hospital screening sample	203	1	1	1	?
	E5939	2009	NLD	Hospital screening sample	78	1	1	1	?
	E5942	2009	NLD	Hospital screening sample	78	1	1	1	?
	UW6455	2005	DEU	Hospital screening sample	192	1	1	1	?
	UW6474	2006	DEU	Hospital screening sample	78	1	1	1	?
	E6419	2010	CHE	Hospital screening sample	203	1	1	1	?
	E6420	2010	CHE	Hospital screening sample	203	1	1	1	?
	E6421	2010	CHE	Hospital screening sample	203	1	1	1	?
	E6422	2010	CHE	Hospital screening sample	203	1	1	1	?
	E6423	2010	CHE	Hospital screening sample	203	1	1	1	?
	E6570	2010	CHE	Hospital screening sample	78	1	1	1	?
	E6424	2010	CHE	Hospital screening sample	192	1	1	1	?
	E6425	2010	CHE	Hospital screening sample	192	1	1	1	?
	E6719	2010	CHE	Hospital screening sample	78	1	1	1	?
	E6956	2010	PRT	Hospital screening sample	78	1	1	1	?
	E7227	2010	GRC	Hospital screening sample	203	1	1	1	?
	E7260	2009	GRC	Hospital screening sample	125	1	1	1	?
	V110	2013	DNK	Hospital screening sample	192	1	1	1	?
	V93	2013	DNK	Hospital screening sample	192	1	1	1	?
	V107	2013	DNK	Hospital screening sample	192	1	1	1	?
	V78	2013	DNK	Hospital screening sample	192	1	1	1	?

V79	2013	DNK	Hospital screening sample	192	1	1	1	?
V80	2013	DNK	Hospital screening sample	192	1	1	1	?
V87	2013	DNK	Hospital screening sample	192	1	1	1	?
V81	2013	DNK	Hospital screening sample	192	1	1	1	?
V83	2013	DNK	Hospital screening sample	192	1	1	1	?
V84	2013	DNK	Hospital screening sample	192	1	1	1	?
V85	2013	DNK	Hospital screening sample	192	1	1	1	?
E5562	2009	CHE	Hospital screening sample	78	1	1	1	?
E5160	2009	CHE	Hospital screening sample	78	1	1	1	?
E6984	2010	LVA	Hospital screening sample	17	1	1	1	?
E6874	2010	CHE	Hospital screening sample	192	1	1	1	?
V86	2013	DNK	Hospital screening sample	192	1	1	1	?
V91	2013	DNK	Hospital screening sample	192	1	1	1	?
V77	2013	DNK	Hospital screening sample	192	1	1	1	?
V94	2013	DNK	Hospital screening sample	192	1	1	1	?
E6698	2010	CHE	Hospital screening sample	361	1	1	1	?
UW5427	2003	DEU	Hospital screening sample	78	1	1	1	?
E6086	2005	DEU	Hospital screening sample	203	1	1	1	*vanA*
E9330	2005	DEU	Hospital screening sample	203	1	1	1	*vanA*
E7377	2012	NLD	Hospital screening sample	78	1	1	1	*vanA*
E6067	2010	PRT	Hospital screening sample	78	1	1	1	*vanA*
E6063	2010	PRT	Hospital screening sample	78	1	1	1	*vanA*
E6048	2010	PRT	Hospital screening sample	78	1	1	1	*vanA*
E6031	2010	LVA	Hospital screening sample	78	1	1	1	*vanA*
E6022	2010	LVA	Hospital screening sample	549	1	1	1	*vanA*
E6013	2010	LVA	Hospital screening sample	78	1	1	1	*vanA*
E7185	2010	PRT	Hospital screening sample	78	1	1	1	*vanA*
E7184	2009	PRT	Hospital screening sample	78	1	1	1	*vanA*
E7183	2009	PRT	Hospital screening sample	78	1	1	1	*vanA*
E7180	2009	PRT	Hospital screening sample	78	1	1	1	*vanA*
E7189	2010	PRT	Hospital screening sample	78	1	1	1	*vanA*
E7179	2009	PRT	Hospital screening sample	78	1	1	1	*vanA*
E7204	2009	GRC	Hospital screening sample	125	1	1	1	*vanA*
E7192	2010	PRT	Hospital screening sample	78	1	1	1	*vanA*
E7259	2008	GRC	Hospital screening sample	18	1	1	1	*vanA*
E7191	2010	PRT	Hospital screening sample	412	1	1	1	*vanA*
E7209	2009	GRC	Hospital screening sample	412	1	1	1	*vanA*
E7188	2010	PRT	Hospital screening sample	78	1	1	1	*vanA*
E7268	2008	GRC	Hospital screening sample	203	1	1	1	*vanA*
E7221	2009	GRC	Hospital screening sample	125	1	1	1	*vanA*
E7220	2008	GRC	Hospital screening sample	412	1	1	1	*vanA*
E7202	2009	LUX	Hospital screening sample	117	1	1	1	*vanA*
E7270	2009	GRC	Hospital screening sample	192	1	1	1	*vanA*
E7269	2010	GRC	Hospital screening sample	412	1	1	1	*vanA*
E7405	2012	NLD	Hospital screening sample	78	1	1	1	*vanA*
E7366	2012	NLD	Hospital screening sample	78	1	1	1	*vanA*
E7418	2012	NLD	Hospital screening sample	78	1	1	1	*vanA*
E8193	2014	NLD	Hospital screening sample	78	1	1	1	*vanA*

E8398	2015	NLD	Hospital screening sample	192	1	1	1	*vanA*
E0161	1995	USA	Hospital screening sample	16	1	1	1	*vanA*
E6049	2010	PRT	Hospital screening sample	78	1	1	1	*vanA*
E6051	2010	PRT	Hospital screening sample	78	1	1	1	*vanA*
E6054	2010	PRT	Hospital screening sample	78	1	1	1	*vanA*
E6052	2010	PRT	Hospital screening sample	78	1	1	1	*vanA*
E6036	2010	LVA	Hospital screening sample	78	1	1	1	*vanA*
E6034	2010	LVA	Hospital screening sample	549	1	1	1	*vanA*
E6033	2010	LVA	Hospital screening sample	78	1	1	1	*vanA*
E6028	2010	LVA	Hospital screening sample	78	1	1	1	*vanA*
E6027	2010	LVA	Hospital screening sample	548	1	1	1	*vanA*
E6024	2010	LVA	Hospital screening sample	549	1	1	1	*vanA*
E6023	2010	LVA	Hospital screening sample	78	1	1	1	*vanA*
E6021	2010	LVA	Hospital screening sample	549	1	1	1	*vanA*
E6019	2010	LVA	Hospital screening sample	78	1	1	1	*vanA*
E6018	2010	LVA	Hospital screening sample	78	1	1	1	*vanA*
E6016	2010	LVA	Hospital screening sample	78	1	1	1	*vanA*
E6015	2010	LVA	Hospital screening sample	548	1	1	1	*vanA*
E6012	2010	LVA	Hospital screening sample	78	1	1	1	*vanA*
E6011	2010	LVA	Hospital screening sample	548	1	1	1	*vanA*
E6010	2010	LVA	Hospital screening sample	548	1	1	1	*vanA*
E6064	2010	PRT	Hospital screening sample	78	1	1	1	*vanA*
E6977	2010	GRC	Hospital screening sample	412	1	1	1	*vanA*
E6976	2010	GRC	Hospital screening sample	412	1	1	1	*vanA*
E6973	2010	GRC	Hospital screening sample	412	1	1	1	*vanA*
E6972	2010	GRC	Hospital screening sample	412	1	1	1	*vanA*
E6953	2010	PRT	Hospital screening sample	78	1	1	1	*vanA*
E6955	2010	PRT	Hospital screening sample	78	1	1	1	*vanA*
E6957	2010	PRT	Hospital screening sample	78	1	1	1	*vanA*
E6959	2010	PRT	Hospital screening sample	78	1	1	1	*vanA*
E7210	2009	GRC	Hospital screening sample	125	1	1	1	*vanA*
E7213	2009	GRC	Hospital screening sample	203	1	1	1	*vanA*
E7174	2010	LVA	Hospital screening sample	549	1	1	1	*vanA*
E7173	2010	LVA	Hospital screening sample	549	1	1	1	*vanA*
E7222	2009	GRC	Hospital screening sample	412	1	1	1	*vanA*
E7267	2010	GRC	Hospital screening sample	412	1	1	1	*vanA*
E7154	2008	LVA	Hospital screening sample	78	1	1	1	*vanA*
E7266	2010	GRC	Hospital screening sample	412	1	1	1	*vanA*
E7229	2010	GRC	Hospital screening sample	125	1	1	1	*vanA*
E7263	2009	GRC	Hospital screening sample	192	1	1	1	*vanA*
E7230	2010	GRC	Hospital screening sample	125	1	1	1	*vanA*
E7238	2011	GRC	Hospital screening sample	412	1	1	1	*vanA*
E7240	2010	GRC	Hospital screening sample	412	1	1	1	*vanA*
E7257	2010	GRC	Hospital screening sample	412	1	1	1	*vanA*
E7242	2009	GRC	Hospital screening sample	203	1	1	1	*vanA*
E7245	2009	GRC	Hospital screening sample	412	1	1	1	*vanA*
E7248	2009	GRC	Hospital screening sample	412	1	1	1	*vanA*
E7253	2009	GRC	Hospital screening sample	125	1	1	1	*vanA*

E7251	2009	GRC	Hospital screening sample	412	1	1	1	*vanA*
E7378	?	NLD	Hospital screening sample	78	1	1	1	*vanA*
E6995	2010	LVA	Hospital screening sample	389	1	1	1	*vanA*
E6986	2010	LVA	Hospital screening sample	389	1	1	1	*vanA*
E6965	2010	LVA	Hospital screening sample	389	1	1	1	*vanA*
E7004	2010	LVA	Hospital screening sample	389	1	1	1	*vanA*
E6961	2010	LVA	Hospital screening sample	389	1	1	1	*vanA*
E7186	2010	PRT	Hospital screening sample	78	1	1	1	*vanA*
E7207	2008	GRC	Hospital screening sample	17	1	1	1	*vanA*
E6044	2010	LVA	Hospital screening sample	389	1	1	1	*vanA*
E6037	2010	LVA	Hospital screening sample	389	1	1	1	*vanA*
E6035	2010	LVA	Hospital screening sample	17	1	1	1	*vanA*
E6025	2010	LVA	Hospital screening sample	547	1	1	1	*vanA*
E6014	2010	LVA	Hospital screening sample	547	1	1	1	*vanA*
E6009	2010	LVA	Hospital screening sample	547	1	1	1	*vanA*
E7003	2010	LVA	Hospital screening sample	389	1	1	1	*vanA*
E7001	2010	LVA	Hospital screening sample	389	1	1	1	*vanA*
E7000	2010	LVA	Hospital screening sample	389	1	1	1	*vanA*
E6997	2010	LVA	Hospital screening sample	389	1	1	1	*vanA*
E6994	2010	LVA	Hospital screening sample	389	1	1	1	*vanA*
E6993	2010	LVA	Hospital screening sample	389	1	1	1	*vanA*
E6992	2010	LVA	Hospital screening sample	389	1	1	1	*vanA*
E6991	2010	LVA	Hospital screening sample	389	1	1	1	*vanA*
E6990	2010	LVA	Hospital screening sample	389	1	1	1	*vanA*
E6989	2010	LVA	Hospital screening sample	389	1	1	1	*vanA*
E6983	2010	LVA	Hospital screening sample	389	1	1	1	*vanA*
E6982	2010	LVA	Hospital screening sample	389	1	1	1	*vanA*
E6970	2010	LVA	Hospital screening sample	389	1	1	1	*vanA*
E6969	2010	LVA	Hospital screening sample	389	1	1	1	*vanA*
E6968	2010	LVA	Hospital screening sample	389	1	1	1	*vanA*
E6964	2010	LVA	Hospital screening sample	389	1	1	1	*vanA*
E6963	2010	LVA	Hospital screening sample	389	1	1	1	*vanA*
E7178	2009	LVA	Hospital screening sample	389	1	1	1	*vanA*
E7167	2010	LVA	Hospital screening sample	389	1	1	1	*vanA*
E7165	2009	LVA	Hospital screening sample	389	1	1	1	*vanA*
E7162	2010	LVA	Hospital screening sample	17	1	1	1	*vanA*
E7161	2010	LVA	Hospital screening sample	17	1	1	1	*vanA*
E7157	2008	LVA	Hospital screening sample	389	1	1	1	*vanA*
E7156	2008	LVA	Hospital screening sample	389	1	1	1	*vanA*
E7153	2008	LVA	Hospital screening sample	547	1	1	1	*vanA*
E7152	2008	LVA	Hospital screening sample	547	1	1	1	*vanA*
E7256	2009	GRC	Hospital screening sample	412	1	1	1	*vanA*
E7247	2009	GRC	Hospital screening sample	412	1	1	1	*vanA*
E8410	2015	NLD	Hospital screening sample	192	1	1	1	*vanA*
E9334	2009	ESP	Hospital screening sample	203	1	1	1	*vanA*
E7258	2009	GRC	Hospital screening sample	192	1	1	1	*vanA*
E6020	2010	LVA	Hospital screening sample	549	1	1	1	*vanA*
E7190	2010	PRT	Hospital screening sample	78	1	1	1	*vanA*

E7262	2010	GRC	Hospital screening sample	125	1	1	1	*vanA*
E6951	2010	LVA	Hospital screening sample	389	1	1	1	*vanA*
E7151	2008	LVA	Hospital screening sample	547	1	1	1	*vanA*
E7424	2009	NLD	Hospital screening sample	78	1	1	1	*vanB*
E7441	2009	NLD	Hospital screening sample	78	1	1	1	*vanB*
E7425	2009	NLD	Hospital screening sample	78	1	1	1	*vanB*
E8234	2015	NLD	Hospital screening sample	203	1	1	1	*vanB*
E7440	2009	NLD	Hospital screening sample	78	1	1	1	*vanB*
E7361	2012	NLD	Hospital screening sample	117	1	1	1	*vanB*
E7360	2012	NLD	Hospital screening sample	117	1	1	1	*vanB*
E0112	1997	NLD	Community isolate	361	1	1	1	None
9339	2008	NOR	Blood culture isolate	192	1	1	1	?
14944	2008	NOR	Blood culture isolate	203	1	1	1	?
20167	2008	NOR	Blood culture isolate	17	1	1	1	?
20972	2008	NOR	Blood culture isolate	203	1	1	1	?
22135	2008	NOR	Blood culture isolate	192	1	1	1	?
30817	2008	NOR	Blood culture isolate	203	1	1	1	?
33027	2008	NOR	Blood culture isolate	17	1	1	1	?
36783	2008	NOR	Blood culture isolate	192	1	1	1	?
37471	2008	NOR	Blood culture isolate	192	1	1	1	?
54271	2008	NOR	Blood culture isolate	203	1	1	1	?
70927	2008	NOR	Blood culture isolate	17	1	1	1	?
73323	2008	NOR	Blood culture isolate	17	1	1	1	?
79932	2008	NOR	Blood culture isolate	17	1	1	1	?
89364	2008	NOR	Blood culture isolate	203	1	1	1	?
656443	2008	NOR	Blood culture isolate	578	1	1	1	?
657214	2008	NOR	Blood culture isolate	17	1	1	1	?
50108366	2008	NOR	Blood culture isolate	203	1	1	1	?
50117412	2008	NOR	Blood culture isolate	203	1	1	1	?
50182281	2008	NOR	Blood culture isolate	203	1	1	1	?
1410801937	2008	NOR	Blood culture isolate	203	1	1	1	?
1410803517	2008	NOR	Blood culture isolate	17	1	1	1	?
1410810681	2008	NOR	Blood culture isolate	192	1	1	1	?
1410825104	2008	NOR	Blood culture isolate	203	1	1	1	?
10487521	2008	NOR	Blood culture isolate	203	1	1	1	?
29966	2008	NOR	Blood culture isolate	78	1	1	1	?
39254	2008	NOR	Blood culture isolate	17	1	1	1	?
418541	2008	NOR	Blood culture isolate	203	1	1	1	?
E2603	2004	NLD	Blood culture isolate	78	1	1	1	?
et20	2008	NOR	Blood culture isolate	17	1	1	1	?
VRE-10	2004	DNK	Blood culture isolate	17	1	1	1	?
VRE1044	2007	SWE	Blood culture isolate	192	1	1	1	?
VRE1261	2008	SWE	Blood culture isolate	192	1	1	1	?
VSE1036	2007	SWE	Blood culture isolate	192	1	1	1	?
E6852	2010	CHE	Blood culture isolate	78	1	1	1	?
E6692	2010	CHE	Blood culture isolate	78	1	1	1	?
E6690	2010	CHE	Blood culture isolate	192	1	1	1	?
E6075	2009	CHE	Blood culture isolate	203	1	1	1	?

	E6689	2010	CHE	Blood culture isolate	192	1	1	1	?
	E6686	2010	CHE	Blood culture isolate	192	1	1	1	?
	E6685	2010	CHE	Blood culture isolate	78	1	1	1	?
	E6857	2010	CHE	Blood culture isolate	203	1	1	1	?
	DO	1992	USA	Blood culture isolate	18	1	1	1	?
	E5188	2009	CHE	Blood culture isolate	78	1	1	1	?
	V45	2012	DNK	Blood culture isolate	192	1	1	1	?
	E1308	?	PRT	Blood culture isolate	17	1	1	1	None
	E1162	1997	FRA	Blood culture isolate	17	1	1	1	None
	E1504	1995	ESP	Blood culture isolate	17	1	1	1	None
	E7556	2012	NLD	Blood culture isolate	78	1	1	1	*vanA*
	E9329	2004	DEU	Blood culture isolate	192	1	1	1	*vanA*
	E9291	2009	ESP	Blood culture isolate	17	1	1	1	*vanA*
	E9296	2001-03	ITA	Blood culture isolate	78	1	1	1	*vanA*
	E7422	2010	NLD	Blood culture isolate	117	1	1	1	*vanB*
**Positive for TirE, lacking Hp1**	VRE-84	2008	DNK	Hospital screening sample	17	1	0	1	?
	E0155	1995	USA	Hospital screening sample	17	1	0	1	*vanA*
	E7607	2013	NLD	Hospital screening sample	192	1	0	1	*vanB*
	E7426	2012	NLD	Hospital screening sample	17	1	0	1	*vanB*
	E7427	2010	NLD	Hospital screening sample	17	1	0	1	*vanB*
	E7438	2010	NLD	Hospital screening sample	17	1	0	1	*vanB*
	E7439	2010	NLD	Hospital screening sample	17	1	0	1	*vanB*
	E7679	2013	NLD	Hospital screening sample	192	1	0	1	*vanB*
	E7676	2013	NLD	Hospital screening sample	192	1	0	1	*vanB*
	E7874	2013	NLD	Hospital screening sample	192	1	0	1	*vanB*
	E7680	2013	NLD	Hospital screening sample	192	1	0	1	*vanB*
	E7682	2013	NLD	Hospital screening sample	192	1	0	1	*vanB*
	E7681	2013	NLD	Hospital screening sample	192	1	0	1	*vanB*
	E7611	2013	NLD	Hospital screening sample	192	1	0	1	*vanB*
	E7612	2013	NLD	Hospital screening sample	192	1	0	1	*vanB*
	E7613	2013	NLD	Hospital screening sample	192	1	0	1	*vanB*
	E7640	2013	NLD	Hospital screening sample	192	1	0	1	*vanB*
	E7643	2013	NLD	Hospital screening sample	192	1	0	1	*vanB*
	E7644	2013	NLD	Hospital screening sample	192	1	0	1	*vanB*
	E7683	2013	NLD	Hospital screening sample	192	1	0	1	*vanB*
	E7687	2013	NLD	Hospital screening sample	192	1	0	1	*vanB*
	E7645	2013	NLD	Hospital screening sample	192	1	0	1	*vanB*
	E7646	2013	NLD	Hospital screening sample	192	1	0	1	*vanB*
	E7694	2013	NLD	Hospital screening sample	192	1	0	1	*vanB*
	E7647	2013	NLD	Hospital screening sample	192	1	0	1	*vanB*
	E7648	2013	NLD	Hospital screening sample	192	1	0	1	*vanB*
	E7649	2013	NLD	Hospital screening sample	192	1	0	1	*vanB*
	E7695	2013	NLD	Hospital screening sample	192	1	0	1	*vanB*
	E7650	2013	NLD	Hospital screening sample	192	1	0	1	*vanB*
	E7651	2013	NLD	Hospital screening sample	192	1	0	1	*vanB*
	E7652	2013	NLD	Hospital screening sample	192	1	0	1	*vanB*
	E7653	2013	NLD	Hospital screening sample	192	1	0	1	*vanB*
	E7654	2013	NLD	Hospital screening sample	192	1	0	1	*vanB*

E7655	2013	NLD	Hospital screening sample	192	1	0	1	*vanB*
E7656	2013	NLD	Hospital screening sample	192	1	0	1	*vanB*
E7657	2013	NLD	Hospital screening sample	192	1	0	1	*vanB*
E7658	2013	NLD	Hospital screening sample	192	1	0	1	*vanB*
E7663	2013	NLD	Hospital screening sample	192	1	0	1	*vanB*
E7664	2013	NLD	Hospital screening sample	192	1	0	1	*vanB*
E7665	2013	NLD	Hospital screening sample	192	1	0	1	*vanB*
E7693	2013	NLD	Hospital screening sample	192	1	0	1	*vanB*
E7666	2013	NLD	Hospital screening sample	192	1	0	1	*vanB*
E7667	2013	NLD	Hospital screening sample	192	1	0	1	*vanB*
E7668	2013	NLD	Hospital screening sample	192	1	0	1	*vanB*
E7669	2013	NLD	Hospital screening sample	192	1	0	1	*vanB*
E7670	2013	NLD	Hospital screening sample	192	1	0	1	*vanB*
E7671	2013	NLD	Hospital screening sample	192	1	0	1	*vanB*
E7677	2013	NLD	Hospital screening sample	192	1	0	1	*vanB*
E7674	2013	NLD	Hospital screening sample	192	1	0	1	*vanB*
E7675	2013	NLD	Hospital screening sample	192	1	0	1	*vanB*
E7701	2013	NLD	Hospital screening sample	192	1	0	1	*vanB*
E7702	2013	NLD	Hospital screening sample	192	1	0	1	*vanB*
E7704	2013	NLD	Hospital screening sample	192	1	0	1	*vanB*
E7705	2013	NLD	Hospital screening sample	192	1	0	1	*vanB*
E7706	2013	NLD	Hospital screening sample	192	1	0	1	*vanB*
E7708	2013	NLD	Hospital screening sample	192	1	0	1	*vanB*
E7709	2013	NLD	Hospital screening sample	192	1	0	1	*vanB*
E7710	2013	NLD	Hospital screening sample	192	1	0	1	*vanB*
E7711	2013	NLD	Hospital screening sample	78	1	0	1	*vanB*
E7833	2013	NLD	Hospital screening sample	192	1	0	1	*vanB*
E7839	2013	NLD	Hospital screening sample	192	1	0	1	*vanB*
E7857	2013	NLD	Hospital screening sample	192	1	0	1	*vanB*
E7954	2013	NLD	Hospital screening sample	192	1	0	1	*vanB*
E8434	2015	NLD	Hospital screening sample	117	1	0	1	*vanB*
E8435	2015	NLD	Hospital screening sample	117	1	0	1	*vanB*
E8445	2015	NLD	Hospital screening sample	117	1	0	1	*vanB*
E8443	2015	NLD	Hospital screening sample	117	1	0	1	*vanB*
E8460	2015	NLD	Hospital screening sample	117	1	0	1	*vanB*
E9012	2015	NLD	Hospital screening sample	117	1	0	1	*vanB*
E7436	2012	NLD	Hospital screening sample	17	1	0	1	*vanB*
E7431	2011	NLD	Hospital screening sample	17	1	0	1	*vanB*
E8412	2015	NLD	Hospital screening sample	117	1	0	1	*VanB*
E7707	2013	NLD	Hospital screening sample	192	1	0	1	*vanB*
E7659	2013	NLD	Hospital screening sample	192	1	0	1	*vanB*
E7856	2013	NLD	Hospital screening sample	192	1	0	1	*vanB*
E7610	2013	NLD	Hospital screening sample	192	1	0	1	*vanB*
E7590	2012	NLD	Hospital screening sample	17	1	0	1	*vanB*
E7642	2013	NLD	Hospital screening sample	192	1	0	1	*vanB*
E7952	2013	NLD	Hospital screening sample	117	1	0	1	*vanB*
10527341	2008	NOR	Blood culture isolate	38	1	0	1	?
50154026	2008	NOR	Blood culture isolate	78	1	0	1	?

	E1193	?	ESP	Blood culture isolate	17	1	0	1	None
**Positive for only 1 or 2**	908301990	2008	NOR	Blood culture isolate	202	1	0	0	?
	U0106/04-535869, X0958	2002	NLD	Blood culture isolate	17	1	0	0	?
	651578	2008	NOR	Blood culture isolate	203	0	1	1	?
	U0262/X2209/04-575201-01	2002	NLD	Blood culture isolate	78	0	1	1	?
	62843	2008	NOR	Blood culture isolate	17	1	1	0	?
	50691662	2013	NOR	Hospital screening sample	unknwon	1	1	0	?
	1410809400	2008	NOR	Blood culture isolate	17	1	1	0	?
	5977	2008	NOR	Blood culture isolate	203	1	1	0	?
	5282	2008	NOR	Blood culture isolate	575	0	1	0	?
**Negative for all 3**	50673722	2012	NOR	Hospital screening sample	872	0	0	0	?
	50701700	2013	NOR	Hospital screening sample	unknwon	0	0	0	?
	50708494	2013	USA	Hospital screening sample	unknwon	0	0	0	?
	A0884	not available	USA	Hospital screening sample	313	0	0	0	?
	A0885	1996	USA	Hospital screening sample	308	0	0	0	?
	E0013	1992	GBR	Hospital screening sample	18	0	0	0	?
	E0073	1995	NLD	Hospital screening sample	22	0	0	0	?
	E0125	1995	NLD	Hospital screening sample	21	0	0	0	?
	E0300	1994	USA	Hospital screening sample	20	0	0	0	?
	E0510	1998	AUS	Hospital screening sample	17	0	0	0	?
	E0745	2000	NLD	Hospital screening sample	16	0	0	0	?
	E1173	not available	PRT	Hospital screening sample	125	0	0	0	?
	E1272	not available	FRA	Hospital screening sample	22	0	0	0	?
	E1279	not available	DEU	Hospital screening sample	117	0	0	0	?
	E1304	not available	PRT	Hospital screening sample	132	0	0	0	?
	E1652	2002	NLD	Hospital screening sample	18	0	0	0	?
	NIZP292/02	2002	POL	Hospital screening sample	17	0	0	0	?
	TUH 2-18	1996	NOR	Hospital screening sample	17	0	0	0	?
	TUH 7-15	not available	USA	Hospital screening sample	16	0	0	0	?
	TUH 7-55	not available	DEU	Hospital screening sample	17	0	0	0	?
	E2036	2000	DEU	Hospital screening sample	17	0	0	0	?
	V305	2013	DNK	Hospital screening sample	80	0	0	0	?
	V28	2013	DNK	Hospital screening sample	80	0	0	0	?
	E5756	2009	NLD	Hospital screening sample	1191	0	0	0	?
	E5757	2009	NLD	Hospital screening sample	540	0	0	0	?
	E5758	2009	NLD	Hospital screening sample	1191	0	0	0	?
	UW5445	2004	DEU	Hospital screening sample	18	0	0	0	?
	E5549	2009	CHE	Hospital screening sample	1189	0	0	0	?
	E5273	2009	CHE	Hospital screening sample	240	0	0	0	?
	E5271	2009	CHE	Hospital screening sample	240	0	0	0	?
	E6709	2010	CHE	Hospital screening sample	22	0	0	0	?
	E6514	2010	CHE	Hospital screening sample	78	0	0	0	?
	E5760	2009	NLD	Hospital screening sample	540	0	0	0	?
	E5776	2009	NLD	Hospital screening sample	1103	0	0	0	?
	E5777	2009	NLD	Hospital screening sample	1103	0	0	0	?
	E6043	2010	LVA	Hospital screening sample	17	0	0	0	?
	E5778	2009	NLD	Hospital screening sample	1103	0	0	0	?

E5779	2009	NLD	Hospital screening sample	1103	0	0	0	?
E5862	2009	NLD	Hospital screening sample	117	0	0	0	?
UW6452	2005	DEU	Hospital screening sample	18	0	0	0	?
E5863	2009	NLD	Hospital screening sample	117	0	0	0	?
E5864	2009	NLD	Hospital screening sample	117	0	0	0	?
E5865	2009	NLD	Hospital screening sample	117	0	0	0	?
E5866	2009	NLD	Hospital screening sample	78	0	0	0	?
E5940	2009	NLD	Hospital screening sample	117	0	0	0	?
E5941	2009	NLD	Hospital screening sample	78	0	0	0	?
E5954	2009	NLD	Hospital screening sample	1103	0	0	0	?
UW6454	2005	DEU	Hospital screening sample	18	0	0	0	?
E5955	2009	NLD	Hospital screening sample	1103	0	0	0	?
E5956	2009	NLD	Hospital screening sample	1103	0	0	0	?
E5957	2009	NLD	Hospital screening sample	1103	0	0	0	?
E5958	2009	NLD	Hospital screening sample	1103	0	0	0	?
E5992	2009	NLD	Hospital screening sample	78	0	0	0	?
E5993	2009	NLD	Hospital screening sample	117	0	0	0	?
E5994	2009	NLD	Hospital screening sample	117	0	0	0	?
E5995	2009	NLD	Hospital screening sample	78	0	0	0	?
E5996	2009	NLD	Hospital screening sample	78	0	0	0	?
E6003	2009	NLD	Hospital screening sample	1103	0	0	0	?
UW6453	2005	DEU	Hospital screening sample	18	0	0	0	?
E5715	2009	NLD	Hospital screening sample	117	0	0	0	?
E5714	2009	NLD	Hospital screening sample	117	0	0	0	?
E6004	2009	NLD	Hospital screening sample	1103	0	0	0	?
E6005	2009	NLD	Hospital screening sample	1103	0	0	0	?
E6006	2009	NLD	Hospital screening sample	1103	0	0	0	?
E6007	2009	NLD	Hospital screening sample	1103	0	0	0	?
UW6456	2005	DEU	Hospital screening sample	18	0	0	0	?
UW6457	2005	DEU	Hospital screening sample	18	0	0	0	?
UW6458	2005	DEU	Hospital screening sample	18	0	0	0	?
UW6459	2005	DEU	Hospital screening sample	18	0	0	0	?
UW6460	2005	DEU	Hospital screening sample	17	0	0	0	?
UW6461	2005	DEU	Hospital screening sample	18	0	0	0	?
UW6463	2006	DEU	Hospital screening sample	18	0	0	0	?
UW6464	2006	DEU	Hospital screening sample	18	0	0	0	?
UW6465	2006	DEU	Hospital screening sample	18	0	0	0	?
UW6466	2006	DEU	Hospital screening sample	18	0	0	0	?
UW6467	2006	DEU	Hospital screening sample	18	0	0	0	?
UW6468	2006	DEU	Hospital screening sample	17	0	0	0	?
UW6469	2006	DEU	Hospital screening sample	18	0	0	0	?
UW6470	2006	DEU	Hospital screening sample	18	0	0	0	?
UW6472	2006	DEU	Hospital screening sample	18	0	0	0	?
UW6473	2006	DEU	Hospital screening sample	17	0	0	0	?
UW6475	2005	DEU	Hospital screening sample	18	0	0	0	?
UW5443	2004	DEU	Hospital screening sample	18	0	0	0	?
UW5442	2004	DEU	Hospital screening sample	18	0	0	0	?
UW5441	2004	DEU	Hospital screening sample	18	0	0	0	?

UW5440	2004	DEU	Hospital screening sample	16	0	0	0	?
UW5438	2004	DEU	Hospital screening sample	18	0	0	0	?
UW5437	2004	DEU	Hospital screening sample	18	0	0	0	?
UW5436	2003	DEU	Hospital screening sample	18	0	0	0	?
UW5435	2004	DEU	Hospital screening sample	18	0	0	0	?
UW5431	2004	DEU	Hospital screening sample	18	0	0	0	?
UW5429	2004	DEU	Hospital screening sample	18	0	0	0	?
UW5428	2004	DEU	Hospital screening sample	18	0	0	0	?
UW6476	2006	DEU	Hospital screening sample	18	0	0	0	?
UW6478	2006	DEU	Hospital screening sample	18	0	0	0	?
UW6480	2006	DEU	Hospital screening sample	17	0	0	0	?
E5711	2009	NLD	Hospital screening sample	117	0	0	0	?
E5712	2009	NLD	Hospital screening sample	117	0	0	0	?
E6715	2010	CHE	Hospital screening sample	1193	0	0	0	?
E5713	2009	NLD	Hospital screening sample	117	0	0	0	?
E5551	2009	CHE	Hospital screening sample	1189	0	0	0	?
E5550	2009	CHE	Hospital screening sample	1189	0	0	0	?
E5025	2009	NLD	Hospital screening sample	1103	0	0	0	?
E5026	2009	NLD	Hospital screening sample	1103	0	0	0	?
E5028	2009	NLD	Hospital screening sample	1103	0	0	0	?
E5548	2009	CHE	Hospital screening sample	1189	0	0	0	?
E5029	2009	NLD	Hospital screening sample	1103	0	0	0	?
E5547	2009	CHE	Hospital screening sample	1189	0	0	0	?
E5431	2009	CHE	Hospital screening sample	78	0	0	0	?
E5331	2009	CHE	Hospital screening sample	18	0	0	0	?
E5274	2009	CHE	Hospital screening sample	178	0	0	0	?
E5272	2009	CHE	Hospital screening sample	1190	0	0	0	?
E5217	2009	CHE	Hospital screening sample	1189	0	0	0	?
E5218	2009	CHE	Hospital screening sample	1051	0	0	0	?
E5262	2009	CHE	Hospital screening sample	22	0	0	0	?
E6194	2009	CHE	Hospital screening sample	117	0	0	0	?
E6251	2009	CHE	Hospital screening sample	117	0	0	0	?
E6519	2010	CHE	Hospital screening sample	78	0	0	0	?
E6437	2010	CHE	Hospital screening sample	117	0	0	0	?
E6512	2010	CHE	Hospital screening sample	78	0	0	0	?
E6716	2010	CHE	Hospital screening sample	21	0	0	0	?
E6718	2010	CHE	Hospital screening sample	21	0	0	0	?
E6728	2010	CHE	Hospital screening sample	117	0	0	0	?
E6859	2010	CHE	Hospital screening sample	271	0	0	0	?
E6860	2010	CHE	Hospital screening sample	271	0	0	0	?
E6864	2010	CHE	Hospital screening sample	18	0	0	0	?
E6869	2010	CHE	Hospital screening sample	78	0	0	0	?
E6879	2010	CHE	Hospital screening sample	192	0	0	0	?
E6884	2010	CHE	Hospital screening sample	78	0	0	0	?
E6889	2010	CHE	Hospital screening sample	78	0	0	0	?
E6952	2010	ITA	Hospital screening sample	74	0	0	0	?
E7224	2009	GRC	Hospital screening sample	203	0	0	0	?
E7264	2009	GRC	Hospital screening sample	286	0	0	0	?

E7234	2010	GRC	Hospital screening sample	17	0	0	0	?
E7249	2009	GRC	Hospital screening sample	17	0	0	0	?
E5609	2009	NLD	Hospital screening sample	?	0	0	0	?
E4954	2009	NLD	Hospital screening sample	?	0	0	0	?
E4824	2009	NLD	Hospital screening sample	18	0	0	0	?
V36	2013	DNK	Hospital screening sample	80	0	0	0	?
V18	2013	DNK	Hospital screening sample	80	0	0	0	?
E4703	2009	NLD	Hospital screening sample	17	0	0	0	?
E5611	2009	NLD	Hospital screening sample	?	0	0	0	?
E6967	2010	LVA	Hospital screening sample	440	0	0	0	?
E5610	2009	NLD	Hospital screening sample	?	0	0	0	?
E4953	2009	NLD	Hospital screening sample	?	0	0	0	?
E4959	2009	NLD	Hospital screening sample	?	0	0	0	?
E5759	2009	NLD	Hospital screening sample	32	0	0	0	?
E5829	2009	NLD	Hospital screening sample	18	0	0	0	?
E5828	2009	NLD	Hospital screening sample	18	0	0	0	?
E5830	2009	NLD	Hospital screening sample	18	0	0	0	?
E5831	2009	NLD	Hospital screening sample	18	0	0	0	?
E5943	2009	NLD	Hospital screening sample	117	0	0	0	?
E4955	2009	NLD	Hospital screening sample	?	0	0	0	?
E4956	2009	NLD	Hospital screening sample	?	0	0	0	?
E4957	2009	NLD	Hospital screening sample	?	0	0	0	?
E4958	2009	NLD	Hospital screening sample	?	0	0	0	?
UW5439	2004	DEU	Hospital screening sample	18	0	0	0	?
E4947	2009	NLD	Hospital screening sample	?	0	0	0	?
E4946	2009	NLD	Hospital screening sample	?	0	0	0	?
UW6481	2006	DEU	Hospital screening sample	18	0	0	0	?
E4945	2009	NLD	Hospital screening sample	?	0	0	0	?
E4944	2009	NLD	Hospital screening sample	?	0	0	0	?
E4943	2009	NLD	Hospital screening sample	?	0	0	0	?
E4827	2009	NLD	Hospital screening sample	18	0	0	0	?
E4826	2009	NLD	Hospital screening sample	18	0	0	0	?
E4825	2009	NLD	Hospital screening sample	18	0	0	0	?
E4823	2009	NLD	Hospital screening sample	18	0	0	0	?
E4770	2009	NLD	Hospital screening sample	17	0	0	0	?
E4769	2009	NLD	Hospital screening sample	17	0	0	0	?
E4768	2009	NLD	Hospital screening sample	17	0	0	0	?
E4767	2009	NLD	Hospital screening sample	17	0	0	0	?
E4766	2009	NLD	Hospital screening sample	17	0	0	0	?
E4696	2009	NLD	Hospital screening sample	17	0	0	0	?
E4705	2009	NLD	Hospital screening sample	17	0	0	0	?
E4697	2009	NLD	Hospital screening sample	17	0	0	0	?
E4698	2009	NLD	Hospital screening sample	17	0	0	0	?
E4704	2009	NLD	Hospital screening sample	17	0	0	0	?
E4699	2009	NLD	Hospital screening sample	17	0	0	0	?
E4700	2009	NLD	Hospital screening sample	17	0	0	0	?
E4701	2009	NLD	Hospital screening sample	17	0	0	0	?
E4702	2009	NLD	Hospital screening sample	17	0	0	0	?

E4960	2009	NLD	Hospital screening sample	?	0	0	0	?
E4961	2009	NLD	Hospital screening sample	?	0	0	0	?
E4962	2009	NLD	Hospital screening sample	?	0	0	0	?
E5027	2009	NLD	Hospital screening sample	?	0	0	0	?
E5039	2009	NLD	Hospital screening sample	?	0	0	0	?
E5040	2009	NLD	Hospital screening sample	?	0	0	0	?
E5042	2009	NLD	Hospital screening sample	?	0	0	0	?
E5043	2009	NLD	Hospital screening sample	?	0	0	0	?
E5268	2009	CHE	Hospital screening sample	38	0	0	0	?
E7176	2009	SVN	Hospital screening sample	279	0	0	0	?
V318	2013	DNK	Hospital screening sample	80	0	0	0	?
V35	2013	DNK	Hospital screening sample	80	0	0	0	?
V298	2013	DNK	Hospital screening sample	80	0	0	0	?
V19	2013	DNK	Hospital screening sample	80	0	0	0	?
V16	2013	DNK	Hospital screening sample	80	0	0	0	?
V15	2013	DNK	Hospital screening sample	80	0	0	0	?
V14	2013	DNK	Hospital screening sample	80	0	0	0	?
V66	2013	DNK	Hospital screening sample	80	0	0	0	?
V102	2013	DNK	Hospital screening sample	80	0	0	0	?
V76	2013	DNK	Hospital screening sample	80	0	0	0	?
V9	2013	DNK	Hospital screening sample	80	0	0	0	?
V88	2013	DNK	Hospital screening sample	80	0	0	0	?
V8	2013	DNK	Hospital screening sample	80	0	0	0	?
E7228	2010	GRC	Hospital screening sample	722	0	0	0	?
E5774	2009	NLD	Hospital screening sample	693	0	0	0	?
E5775	2009	NLD	Hospital screening sample	693	0	0	0	?
E5772	2009	NLD	Hospital screening sample	693	0	0	0	?
E5771	2009	NLD	Hospital screening sample	693	0	0	0	?
E5773	2009	NLD	Hospital screening sample	693	0	0	0	?
E5030	2009	NLD	Hospital screening sample	693	0	0	0	?
E5031	2009	NLD	Hospital screening sample	693	0	0	0	?
E5032	2009	NLD	Hospital screening sample	693	0	0	0	?
E5033	2009	NLD	Hospital screening sample	693	0	0	0	?
E6894	2010	CHE	Hospital screening sample	994	0	0	0	?
E6511	2010	CHE	Hospital screening sample	1192	0	0	0	?
E6301	2009	CHE	Hospital screening sample	240	0	0	0	?
E6426	2010	CHE	Hospital screening sample	240	0	0	0	?
V37	2013	DNK	Hospital screening sample	80	0	0	0	?
E6998	2010	LVA	Hospital screening sample	642	0	0	0	?
UW5446	2004	DEU	Hospital screening sample	18	0	0	0	?
UW5447	2004	DEU	Hospital screening sample	16	0	0	0	?
UW5434	2004	DEU	Hospital screening sample	18	0	0	0	?
UW5433	2004	DEU	Hospital screening sample	16	0	0	0	?
V52	2013	DNK	Hospital screening sample	80	0	0	0	?
V13	2012	DNK	Hospital screening sample	80	0	0	0	?
UW5444	2004	DEU	Hospital screening sample	18	0	0	0	?
E1321	1999	ITA	Hospital screening sample	78	0	0	0	None
E8456	2015	NLD	Hospital screening sample	205	0	0	0	None

E1677	?	BRA	Hospital screening sample	113	0	0	0	None
E1669	?	BRA	Hospital screening sample	94	0	0	0	None
E1172	?	POL	Hospital screening sample	384	0	0	0	None
E7574	2012	NLD	Hospital screening sample	117	0	0	0	None
E7500	2012	NLD	Hospital screening sample	203	0	0	0	*vanA*
E7885	2013	NLD	Hospital screening sample	203	0	0	0	*vanA*
E7573	2012	NLD	Hospital screening sample	203	0	0	0	*vanA*
E8233	2015	NLD	Hospital screening sample	203	0	0	0	*vanA*
E9328	1998	DEU	Hospital screening sample	117	0	0	0	*vanA*
E8045	2014	NLD	Hospital screening sample	17	0	0	0	*vanA*
E9287	2003	ESP	Hospital screening sample	17	0	0	0	*vanA*
E1680	1998	BRA	Hospital screening sample	114	0	0	0	*vanA*
E7211	2009	GRC	Hospital screening sample	17	0	0	0	*vanA*
E7521	2012	NLD	Hospital screening sample	290	0	0	0	*vanA*
E7518	2012	NLD	Hospital screening sample	290	0	0	0	*vanA*
E7931	2013	NLD	Hospital screening sample	203	0	0	0	*vanA*
E8224	2015	NLD	Hospital screening sample	203	0	0	0	*vanA*
E8455	2015	NLD	Hospital screening sample	17	0	0	0	*vanA*
E6066	2010	PRT	Hospital screening sample	117	0	0	0	*vanA*
E6058	2010	PRT	Hospital screening sample	117	0	0	0	*vanA*
E6061	2010	PRT	Hospital screening sample	551	0	0	0	*vanA*
E7194	2010	PRT	Hospital screening sample	125	0	0	0	*vanA*
E6988	2010	LVA	Hospital screening sample	17	0	0	0	*vanA*
E7198	2009	LUX	Hospital screening sample	80	0	0	0	*vanA*
E7203	2009	GRC	Hospital screening sample	552	0	0	0	*vanA*
E7200	2009	LUX	Hospital screening sample	78	0	0	0	*vanA*
E7196	2011	FRA	Hospital screening sample	656	0	0	0	*vanA*
E7187	2010	PRT	Hospital screening sample	721	0	0	0	*vanA*
E7225	2009	GRC	Hospital screening sample	203	0	0	0	*vanA*
E7216	2009	GRC	Hospital screening sample	17	0	0	0	*vanA*
E7261	2010	GRC	Hospital screening sample	17	0	0	0	*vanA*
E7201	2009	LUX	Hospital screening sample	78	0	0	0	*vanA*
E7254	2009	GRC	Hospital screening sample	192	0	0	0	*vanA*
E7252	2009	GRC	Hospital screening sample	203	0	0	0	*vanA*
E7432	2012	NLD	Hospital screening sample	1177	0	0	0	*vanA*
E7555	2012	NLD	Hospital screening sample	203	0	0	0	*vanA*
E7513	2012	NLD	Hospital screening sample	203	0	0	0	*vanA*
E7419	2012	NLD	Hospital screening sample	290	0	0	0	*vanA*
E7477	2012	NLD	Hospital screening sample	736	0	0	0	*vanA*
E7884	2013	NLD	Hospital screening sample	203	0	0	0	*vanA*
E7685	2013	NLD	Hospital screening sample	185	0	0	0	*vanA*
E7495	2012	NLD	Hospital screening sample	290	0	0	0	*vanA*
E7496	2012	NLD	Hospital screening sample	290	0	0	0	*vanA*
E7497	2012	NLD	Hospital screening sample	290	0	0	0	*vanA*
E7498	2012	NLD	Hospital screening sample	290	0	0	0	*vanA*
E7504	2012	NLD	Hospital screening sample	203	0	0	0	*vanA*
E7520	2012	NLD	Hospital screening sample	290	0	0	0	*vanA*
E7876	2013	NLD	Hospital screening sample	203	0	0	0	*vanA*

E7886	2013	NLD	Hospital screening sample	203	0	0	0	*vanA*
E7641	2013	NLD	Hospital screening sample	117	0	0	0	*vanA*
E7684	2013	NLD	Hospital screening sample	203	0	0	0	*vanA*
E7877	2013	NLD	Hospital screening sample	203	0	0	0	*vanA*
E7688	2013	NLD	Hospital screening sample	203	0	0	0	*vanA*
E7696	2013	NLD	Hospital screening sample	203	0	0	0	*vanA*
E7697	2013	NLD	Hospital screening sample	203	0	0	0	*vanA*
E7882	2013	NLD	Hospital screening sample	203	0	0	0	*vanA*
E7678	2013	NLD	Hospital screening sample	736	0	0	0	*vanA*
E7672	2013	NLD	Hospital screening sample	203	0	0	0	*vanA*
E7673	2013	NLD	Hospital screening sample	203	0	0	0	*vanA*
E7698	2013	NLD	Hospital screening sample	203	0	0	0	*vanA*
E7699	2013	NLD	Hospital screening sample	203	0	0	0	*vanA*
E7700	2013	NLD	Hospital screening sample	203	0	0	0	*vanA*
E7703	2013	NLD	Hospital screening sample	203	0	0	0	*vanA*
E7836	2013	NLD	Hospital screening sample	203	0	0	0	*vanA*
E7941	2013	NLD	Hospital screening sample	117	0	0	0	*vanA*
E7872	2013	NLD	Hospital screening sample	203	0	0	0	*vanA*
E8014	2014	NLD	Hospital screening sample	203	0	0	0	*vanA*
E7932	2013	NLD	Hospital screening sample	203	0	0	0	*vanA*
E7926	2013	NLD	Hospital screening sample	89	0	0	0	*vanA*
E8020	2014	NLD	Hospital screening sample	203	0	0	0	*vanA*
E8182	2014	NLD	Hospital screening sample	203	0	0	0	*vanA*
E8017	2014	NLD	Hospital screening sample	203	0	0	0	*vanA*
E8013	2014	NLD	Hospital screening sample	203	0	0	0	*vanA*
E8179	2014	NLD	Hospital screening sample	18	0	0	0	*vanA*
E8005	2014	NLD	Hospital screening sample	203	0	0	0	*vanA*
E8001	2014	NLD	Hospital screening sample	203	0	0	0	*vanA*
E8002	2014	NLD	Hospital screening sample	203	0	0	0	*vanA*
E8003	2014	NLD	Hospital screening sample	203	0	0	0	*vanA*
E8180	2014	NLD	Hospital screening sample	911	0	0	0	*vanA*
E8184	2014	NLD	Hospital screening sample	203	0	0	0	*vanA*
E8129	2014	NLD	Hospital screening sample	203	0	0	0	*vanA*
E8185	2014	NLD	Hospital screening sample	203	0	0	0	*vanA*
E8191	2014	NLD	Hospital screening sample	203	0	0	0	*vanA*
E8192	2014	NLD	Hospital screening sample	203	0	0	0	*vanA*
E8189	2014	NLD	Hospital screening sample	203	0	0	0	*vanA*
E8390	2015	NLD	Hospital screening sample	203	0	0	0	*vanA*
E8173	2014	NLD	Hospital screening sample	203	0	0	0	*vanA*
E8200	2015	NLD	Hospital screening sample	18	0	0	0	*vanA*
E8201	2015	NLD	Hospital screening sample	203	0	0	0	*vanA*
E8205	2015	NLD	Hospital screening sample	203	0	0	0	*vanA*
E8210	2015	NLD	Hospital screening sample	203	0	0	0	*vanA*
E8447	2015	NLD	Hospital screening sample	50	0	0	0	*vanA*
E8449	2015	NLD	Hospital screening sample	203	0	0	0	*vanA*
E8448	2015	NLD	Hospital screening sample	50	0	0	0	*vanA*
E8394	2015	NLD	Hospital screening sample	17	0	0	0	*vanA*
E8395	2015	NLD	Hospital screening sample	203	0	0	0	*vanA*

E8397	2015	NLD	Hospital screening sample	80	0	0	0	*vanA*
E8392	2015	NLD	Hospital screening sample	5	0	0	0	*vanA*
E8439	2015	NLD	Hospital screening sample	18	0	0	0	*vanA*
E8393	2015	NLD	Hospital screening sample	17	0	0	0	*vanA*
E8403	2015	NLD	Hospital screening sample	6	0	0	0	*vanA*
E8437	2015	NLD	Hospital screening sample	203	0	0	0	*vanA*
E8442	2015	NLD	Hospital screening sample	203	0	0	0	*vanA*
E8415	2015	NLD	Hospital screening sample	203	0	0	0	*vanA*
E9001	2015	NLD	Hospital screening sample	546	0	0	0	*vanA*
E9015	2015	NLD	Hospital screening sample	5	0	0	0	*vanA*
E0323	1997	FRA	Hospital screening sample	79	0	0	0	*vanA*
E6053	2010	PRT	Hospital screening sample	18	0	0	0	*vanA*
E1132	2001	USA	Hospital screening sample	16	0	0	0	*vanA*
E6057	2010	PRT	Hospital screening sample	117	0	0	0	*vanA*
E6056	2010	PRT	Hospital screening sample	117	0	0	0	*vanA*
E6046	2010	PRT	Hospital screening sample	117	0	0	0	*vanA*
E6032	2010	LVA	Hospital screening sample	17	0	0	0	*vanA*
E6029	2010	LVA	Hospital screening sample	18	0	0	0	*vanA*
E6065	2010	PRT	Hospital screening sample	117	0	0	0	*vanA*
E6062	2010	PRT	Hospital screening sample	117	0	0	0	*vanA*
E6060	2010	PRT	Hospital screening sample	117	0	0	0	*vanA*
E6059	2010	PRT	Hospital screening sample	117	0	0	0	*vanA*
E7002	2010	LVA	Hospital screening sample	117	0	0	0	*vanA*
E6999	2010	LVA	Hospital screening sample	117	0	0	0	*vanA*
E6987	2010	LVA	Hospital screening sample	117	0	0	0	*vanA*
E6980	2010	LVA	Hospital screening sample	89	0	0	0	*vanA*
E6979	2010	GRC	Hospital screening sample	117	0	0	0	*vanA*
E6978	2010	GRC	Hospital screening sample	117	0	0	0	*vanA*
E6975	2010	GRC	Hospital screening sample	203	0	0	0	*vanA*
E7193	2010	PRT	Hospital screening sample	564	0	0	0	*vanA*
E6954	2010	PRT	Hospital screening sample	17	0	0	0	*vanA*
E6958	2010	PRT	Hospital screening sample	280	0	0	0	*vanA*
E7226	2010	GRC	Hospital screening sample	117	0	0	0	*vanA*
E7411	2012	NLD	Hospital screening sample	18	0	0	0	*vanA*
E7182	2009	PRT	Hospital screening sample	117	0	0	0	*vanA*
E7181	2009	PRT	Hospital screening sample	117	0	0	0	*vanA*
E7214	2009	GRC	Hospital screening sample	94	0	0	0	*vanA*
E7172	2010	LVA	Hospital screening sample	18	0	0	0	*vanA*
E7171	2010	LVA	Hospital screening sample	18	0	0	0	*vanA*
E7168	2010	LVA	Hospital screening sample	18	0	0	0	*vanA*
E7271	2008	GRC	Hospital screening sample	117	0	0	0	*vanA*
E7164	2009	LVA	Hospital screening sample	117	0	0	0	*vanA*
E7163	2009	LVA	Hospital screening sample	117	0	0	0	*vanA*
E7219	2009	GRC	Hospital screening sample	17	0	0	0	*vanA*
E7223	2009	GRC	Hospital screening sample	17	0	0	0	*vanA*
E7155	2008	LVA	Hospital screening sample	18	0	0	0	*vanA*
E7265	2009	GRC	Hospital screening sample	17	0	0	0	*vanA*
E7231	2010	GRC	Hospital screening sample	203	0	0	0	*vanA*

E7232	2010	GRC	Hospital screening sample	323	0	0	0	*vanA*
E7392	2012	NLD	Hospital screening sample	209	0	0	0	*vanA*
E7235	2011	GRC	Hospital screening sample	117	0	0	0	*vanA*
E7236	2011	GRC	Hospital screening sample	203	0	0	0	*vanA*
E7239	2010	GRC	Hospital screening sample	17	0	0	0	*vanA*
E7241	2009	GRC	Hospital screening sample	203	0	0	0	*vanA*
E7243	2009	GRC	Hospital screening sample	203	0	0	0	*vanA*
E7244	2009	GRC	Hospital screening sample	192	0	0	0	*vanA*
E7246	2009	GRC	Hospital screening sample	203	0	0	0	*vanA*
E7503	2012	NLD	Hospital screening sample	203	0	0	0	*vanA*
E7319	2012	NLD	Hospital screening sample	203	0	0	0	*vanA*
E7471	2012	NLD	Hospital screening sample	18	0	0	0	*vanA*
E8188	2014	NLD	Hospital screening sample	965	0	0	0	*vanA*
E8049	2014	NLD	Hospital screening sample	117	0	0	0	*vanA*
E8046	2014	NLD	Hospital screening sample	494	0	0	0	*vanA*
E8044	2014	NLD	Hospital screening sample	203	0	0	0	*vanA*
E8035	2014	NLD	Hospital screening sample	203	0	0	0	*vanA*
E7999	2014	NLD	Hospital screening sample	203	0	0	0	*vanA*
E7996	2014	NLD	Hospital screening sample	203	0	0	0	*vanA*
E7995	2014	NLD	Hospital screening sample	203	0	0	0	*vanA*
E7994	2014	NLD	Hospital screening sample	203	0	0	0	*vanA*
E7993	2014	NLD	Hospital screening sample	203	0	0	0	*vanA*
E8011	2014	NLD	Hospital screening sample	203	0	0	0	*vanA*
E7992	2014	NLD	Hospital screening sample	203	0	0	0	*vanA*
E7991	2014	NLD	Hospital screening sample	203	0	0	0	*vanA*
E8012	2014	NLD	Hospital screening sample	323	0	0	0	*vanA*
E7990	2014	NLD	Hospital screening sample	203	0	0	0	*vanA*
E7986	2014	NLD	Hospital screening sample	203	0	0	0	*vanA*
E7968	2013	NLD	Hospital screening sample	203	0	0	0	*vanA*
E8018	2014	NLD	Hospital screening sample	203	0	0	0	*vanA*
E7967	2013	NLD	Hospital screening sample	203	0	0	0	*vanA*
E8019	2013	NLD	Hospital screening sample	80	0	0	0	*vanA*
E7837	2013	NLD	Hospital screening sample	494	0	0	0	*vanA*
E8459	2015	NLD	Hospital screening sample	50	0	0	0	*vanA*
E9005	2015	NLD	Hospital screening sample	911	0	0	0	*vanA*
E9006	2015	NLD	Hospital screening sample	80	0	0	0	*vanA*
E8170	2014	NLD	Hospital screening sample	80	0	0	0	*vanA*
E8175	2014	NLD	Hospital screening sample	80	0	0	0	*vanA*
E6068	2010	PRT	Hospital screening sample	18	0	0	0	*vanA*
E6040	2010	LVA	Hospital screening sample	440	0	0	0	*vanA*
E6962	2010	LVA	Hospital screening sample	440	0	0	0	*vanA*
E7255	2010	GRC	Hospital screening sample	117	0	0	0	*vanA*
E7321	2012	NLD	Hospital screening sample	375	0	0	0	*vanA*
E8387	2015	NLD	Hospital screening sample	80	0	0	0	*vanA*
E7928	2013	NLD	Hospital screening sample	117	0	0	0	*vanA*
E8107	2013	NLD	Hospital screening sample	266	0	0	0	*vanA*
E8106	2013	NLD	Hospital screening sample	266	0	0	0	*vanA*
E8057	2014	NLD	Hospital screening sample	18	0	0	0	*vanA*

E8174	2014	NLD	Hospital screening sample	80	0	0	0	*vanA*
E8167	2014	NLD	Hospital screening sample	80	0	0	0	*vanA*
E8396	2015	NLD	Hospital screening sample	80	0	0	0	*vanA*
E8421	2015	NLD	Hospital screening sample	203	0	0	0	*vanA*
E0005	1986	FRA	Hospital screening sample	25	0	0	0	*vanA*
E0805	2000	NLD	Hospital screening sample	16	0	0	0	*VanA*
E6039	2010	LVA	Hospital screening sample	17	0	0	0	*vanA*
E6041	2010	LVA	Hospital screening sample	440	0	0	0	*vanA*
E6042	2010	LVA	Hospital screening sample	440	0	0	0	*vanA*
E6038	2010	LVA	Hospital screening sample	440	0	0	0	*vanA*
E6030	2010	LVA	Hospital screening sample	440	0	0	0	*vanA*
E6017	2010	LVA	Hospital screening sample	440	0	0	0	*vanA*
E6055	2010	PRT	Hospital screening sample	18	0	0	0	*vanA*
E6985	2010	LVA	Hospital screening sample	17	0	0	0	*vanA*
E6981	2010	LVA	Hospital screening sample	17	0	0	0	*vanA*
E6971	2010	GRC	Hospital screening sample	655	0	0	0	*vanA*
E6966	2010	LVA	Hospital screening sample	17	0	0	0	*vanA*
E7177	2009	SVN	Hospital screening sample	279	0	0	0	*vanA*
E7175	2009	LVA	Hospital screening sample	17	0	0	0	*vanA*
E7170	2010	LVA	Hospital screening sample	440	0	0	0	*vanA*
E7169	2010	LVA	Hospital screening sample	440	0	0	0	*vanA*
E7160	2009	SVN	Hospital screening sample	279	0	0	0	*vanA*
E7159	2009	LVA	Hospital screening sample	440	0	0	0	*vanA*
E7158	2009	LVA	Hospital screening sample	440	0	0	0	*vanA*
E7233	2010	GRC	Hospital screening sample	17	0	0	0	*vanA*
E7393	2012	NLD	Hospital screening sample	117	0	0	0	*vanA*
E7437	2011	NLD	Hospital screening sample	375	0	0	0	*vanA*
E8164	2014	NLD	Hospital screening sample	80	0	0	0	*vanA*
E8158	2014	NLD	Hospital screening sample	80	0	0	0	*vanA*
E8157	2014	NLD	Hospital screening sample	80	0	0	0	*vanA*
E8168	2014	NLD	Hospital screening sample	80	0	0	0	*vanA*
E8156	2014	NLD	Hospital screening sample	80	0	0	0	*vanA*
E8128	2014	NLD	Hospital screening sample	80	0	0	0	*vanA*
E8052	2014	NLD	Hospital screening sample	18	0	0	0	*vanA*
E8048	2014	NLD	Hospital screening sample	80	0	0	0	*vanA*
E8047	2014	NLD	Hospital screening sample	18	0	0	0	*vanA*
E8108	2013	NLD	Hospital screening sample	266	0	0	0	*vanA*
E8034	2014	NLD	Hospital screening sample	18	0	0	0	*vanA*
E8033	2014	NLD	Hospital screening sample	18	0	0	0	*vanA*
E8109	2013	NLD	Hospital screening sample	266	0	0	0	*vanA*
E8027	2014	NLD	Hospital screening sample	18	0	0	0	*vanA*
E8176	2014	NLD	Hospital screening sample	89	0	0	0	*vanA*
E7412	2012	NLD	Hospital screening sample	117	0	0	0	*vanA*
E7609	2013	NLD	Hospital screening sample	89	0	0	0	*vanA*
E1133	2001	USA	Hospital screening sample	117	0	0	0	*VanA*
E8010	2014	NLD	Hospital screening sample	6	0	0	0	*vanA*
E8177	2014	NLD	Hospital screening sample	952	0	0	0	*vanA*
E1141	1995	NLD	Hospital screening sample	123	0	0	0	*vanA*

E1695	1998	BRA	Hospital screening sample	97	0	0	0	*vanA*
E6026	2010	LVA	Hospital screening sample	17	0	0	0	*vanA*
E7516	2012	NLD	Hospital screening sample	290	0	0	0	*vanA*
E7989	2014	NLD	Hospital screening sample	203	0	0	0	*vanA*
E7404	2012	NLD	Hospital screening sample	203	0	0	0	*vanA*
E7964	2013	NLD	Hospital screening sample	203	0	0	0	*vanA*
E0292	1992	USA	Hospital screening sample	20	0	0	0	*vanA*
E0300	1994	USA	Hospital screening sample	20	0	0	0	*vanA*
E9314	2002	PRT	Hospital screening sample	132	0	0	0	*vanA*
E9315	2002-03	PRT	Hospital screening sample	280	0	0	0	*vanA*
E9317	2005	ESP	Hospital screening sample	17	0	0	0	*vanA*
E9321	1997-1999	AUS	Hospital screening sample	16	0	0	0	*vanA*
E7470	2012	NLD	Hospital screening sample	203	0	0	0	*vanA*
E0013	1992	GBR	Hospital screening sample	18	0	0	0	*vanA*
E8155	2014	NLD	Hospital screening sample	80	0	0	0	*vanA*
E7320	2012	NLD	Hospital screening sample	203	0	0	0	*vanA*
E7205	2009	GRC	Hospital screening sample	203	0	0	0	*vanA*
E7858	2013	NLD	Hospital screening sample	203	0	0	0	*vanA*
E0525	1998	AUS	Hospital screening sample	17	0	0	0	*vanA*
E9313	2002	PRT	Hospital screening sample	5	0	0	0	*vanA*
E9333	2008	ESP	Hospital screening sample	117	0	0	0	*vanA*
E7250	2009	GRC	Hospital screening sample	203	0	0	0	*vanA*
E9289	2005	POL	Hospital screening sample	202	0	0	0	*vanA*
E6996	2010	LVA	Hospital screening sample	440	0	0	0	*vanA*
E1651	2002	NLD	Hospital screening sample	16	0	0	0	*VanA*
E7212	2009	GRC	Hospital screening sample	117	0	0	0	*vanA/vanB*
E8453	2015	NLD	Hospital screening sample	117	0	0	0	*vanB*
E7429	2010	NLD	Hospital screening sample	117	0	0	0	*vanB*
E7428	2011	NLD	Hospital screening sample	117	0	0	0	*vanB*
E8166	2014	NLD	Hospital screening sample	117	0	0	0	*vanB*
E7662	2013	NLD	Hospital screening sample	117	0	0	0	*vanB*
E9292	2006	ESP	Hospital screening sample	17	0	0	0	*vanB*
E7348	2012	NLD	Hospital screening sample	117	0	0	0	*vanB*
E7380	2012	NLD	Hospital screening sample	117	0	0	0	*vanB*
E8051	2014	NLD	Hospital screening sample	117	0	0	0	*vanB*
E8115	2013	NLD	Hospital screening sample	290	0	0	0	*vanB*
E7217	2009	GRC	Hospital screening sample	117	0	0	0	*vanB*
E7381	2012	NLD	Hospital screening sample	117	0	0	0	*vanB*
E7383	2012	NLD	Hospital screening sample	117	0	0	0	*vanB*
E7382	2012	NLD	Hospital screening sample	117	0	0	0	*vanB*
E7430	2011	NLD	Hospital screening sample	117	0	0	0	*vanB*
E7352	2012	NLD	Hospital screening sample	117	0	0	0	*vanB*
E7338	2012	NLD	Hospital screening sample	117	0	0	0	*vanB*
E7339	2012	NLD	Hospital screening sample	117	0	0	0	*vanB*
E7340	2012	NLD	Hospital screening sample	117	0	0	0	*vanB*
E7343	2012	NLD	Hospital screening sample	117	0	0	0	*vanB*
E7344	2012	NLD	Hospital screening sample	117	0	0	0	*vanB*

E7362	2012	NLD	Hospital screening sample	117	0	0	0	*vanB*
E7363	2012	NLD	Hospital screening sample	117	0	0	0	*vanB*
E7379	2012	NLD	Hospital screening sample	117	0	0	0	*vanB*
E7367	2012	NLD	Hospital screening sample	117	0	0	0	*vanB*
E7375	2012	NLD	Hospital screening sample	117	0	0	0	*vanB*
E7376	2012	NLD	Hospital screening sample	117	0	0	0	*vanB*
E7947	2013	NLD	Hospital screening sample	80	0	0	0	*vanB*
E7915	2013	NLD	Hospital screening sample	117	0	0	0	*vanB*
E8112	2013	NLD	Hospital screening sample	117	0	0	0	*vanB*
E8113	2013	NLD	Hospital screening sample	117	0	0	0	*vanB*
E8111	2013	NLD	Hospital screening sample	117	0	0	0	*vanB*
E8114	2013	NLD	Hospital screening sample	290	0	0	0	*vanB*
E8183	2014	NLD	Hospital screening sample	117	0	0	0	*vanB*
E8178	2014	NLD	Hospital screening sample	117	0	0	0	*vanB*
E8122	2013	NLD	Hospital screening sample	117	0	0	0	*vanB*
E8123	2013	NLD	Hospital screening sample	117	0	0	0	*vanB*
E8124	2014	NLD	Hospital screening sample	117	0	0	0	*vanB*
E8240	2015	NLD	Hospital screening sample	117	0	0	0	*vanB*
E8131	2014	NLD	Hospital screening sample	117	0	0	0	*vanB*
E8145	2014	NLD	Hospital screening sample	117	0	0	0	*vanB*
E8152	2014	NLD	Hospital screening sample	117	0	0	0	*vanB*
E8153	2014	NLD	Hospital screening sample	117	0	0	0	*vanB*
E8154	2014	NLD	Hospital screening sample	117	0	0	0	*vanB*
E8206	2015	NLD	Hospital screening sample	117	0	0	0	*vanB*
E8285	2015	NLD	Hospital screening sample	117	0	0	0	*vanB*
E8413	2015	NLD	Hospital screening sample	117	0	0	0	*vanB*
E7199	2009	LUX	Hospital screening sample	282	0	0	0	*vanB*
E7218	2009	GRC	Hospital screening sample	117	0	0	0	*vanB*
E7237	2010	GRC	Hospital screening sample	117	0	0	0	*vanB*
E7359	2012	NLD	Hospital screening sample	117	0	0	0	*vanB*
E7358	2012	NLD	Hospital screening sample	117	0	0	0	*vanB*
E7357	2012	NLD	Hospital screening sample	117	0	0	0	*vanB*
E7356	2012	NLD	Hospital screening sample	117	0	0	0	*vanB*
E7355	2012	NLD	Hospital screening sample	117	0	0	0	*vanB*
E7354	2012	NLD	Hospital screening sample	117	0	0	0	*vanB*
E8148	2014	NLD	Hospital screening sample	17	0	0	0	*vanB*
E8130	2014	NLD	Hospital screening sample	117	0	0	0	*vanB*
E8120	2013	NLD	Hospital screening sample	117	0	0	0	*vanB*
E8119	2013	NLD	Hospital screening sample	117	0	0	0	*vanB*
E8118	2013	NLD	Hospital screening sample	117	0	0	0	*vanB*
E8117	2013	NLD	Hospital screening sample	290	0	0	0	*vanB*
E8116	2013	NLD	Hospital screening sample	290	0	0	0	*vanB*
E7948	2013	NLD	Hospital screening sample	80	0	0	0	*vanB*
E9018	2015	NLD	Hospital screening sample	117	0	0	0	*vanB*
E7486	2012	NLD	Hospital screening sample	117	0	0	0	*vanB*
E7444	2012	NLD	Hospital screening sample	18	0	0	0	*vanB*
E7949	2013	NLD	Hospital screening sample	80	0	0	0	*vanB*
E7686	2013	NLD	Hospital screening sample	117	0	0	0	*vanB*

E8110	2013	NLD	Hospital screening sample	117	0	0	0	*vanB*
E8235	2015	NLD	Hospital screening sample	18	0	0	0	*vanB*
E8000	2014	NLD	Hospital screening sample	78	0	0	0	*vanB*
E8062	2014	NLD	Hospital screening sample	117	0	0	0	*vanB*
E8147	2014	NLD	Hospital screening sample	80	0	0	0	*vanB*
E8401	2015	NLD	Hospital screening sample	18	0	0	0	*vanB*
E7166	2010	FRA	Hospital screening sample	1119	0	0	0	*vanB*
E7423	2010	NLD	Hospital screening sample	117	0	0	0	*vanB*
E7353	2012	NLD	Hospital screening sample	117	0	0	0	*vanB*
E7349	2012	NLD	Hospital screening sample	117	0	0	0	*vanB*
E7434	2012	NLD	Hospital screening sample	117	0	0	0	*vanB*
E7591	2013	NLD	Hospital screening sample	117	0	0	0	*vanB*
E7592	2013	NLD	Hospital screening sample	117	0	0	0	*vanB*
E8058	2014	NLD	Hospital screening sample	117	0	0	0	*vanB*
E8059	2014	NLD	Hospital screening sample	117	0	0	0	*vanB*
E8037	2014	NLD	Hospital screening sample	80	0	0	0	*vanB*
E8036	2014	NLD	Hospital screening sample	80	0	0	0	*vanB*
E7953	2013	NLD	Hospital screening sample	80	0	0	0	*vanB*
E7951	2013	NLD	Hospital screening sample	80	0	0	0	*vanB*
E7950	2013	NLD	Hospital screening sample	80	0	0	0	*vanB*
E7689	2013	NLD	Hospital screening sample	117	0	0	0	*vanB*
E7337	2012	NLD	Hospital screening sample	117	0	0	0	*vanB*
E7351	2012	NLD	Hospital screening sample	117	0	0	0	*vanB*
E8121	2013	NLD	Hospital screening sample	117	0	0	0	*vanB*
E9302	2003	ESP	Hospital screening sample	18	0	0	0	*vanB*
E9307	2004	CHI	Hospital screening sample	64	0	0	0	*vanB*
E8330	2015	NLD	Hospital screening sample	117	0	0	0	*vanB*
E9293	1998	ESP	Hospital screening sample	265	0	0	0	*vanB*
E0532	1998	AUS	Hospital screening sample	17	0	0	0	*vanB*
E8263	2015	NLD	Hospital screening sample	117	0	0	0	*vanB*
E7946	2013	NLD	Hospital screening sample	80	0	0	0	*vanB*
E9343	2003	CHI	Hospital screening sample	64	0	0	0	*vanB*
E8163	2014	NLD	Hospital screening sample	117	0	0	0	*vanB*
E8146	2014	NLD	Hospital screening sample	80	0	0	0	*vanB*
E9288	2004	ESP	Hospital screening sample	17	0	0	0	*vanB*
E9242	2016	NLD	Hospital screening sample	262	0	0	0	vanD
50917236	2015	NOR	Community isolate	178	0	0	0	?
50927302	2015	NOR	Community isolate	unknwon	0	0	0	?
50939184	2015	NOR	Community isolate	800	0	0	0	?
50941940	2015	NOR	Community isolate	580	0	0	0	?
50945943	2015	NOR	Community isolate	unknown	0	0	0	?
50945961	2015	NOR	Community isolate	22	0	0	0	?
50945964	2015	NOR	Community isolate	unknwon	0	0	0	?
50933849-1	2015	NOR	Community isolate	unknwon	0	0	0	?
A0248	1999	NOR	Community isolate	195	0	0	0	?
A0889	1998	NOR	Community isolate	312	0	0	0	?
E0092	1997	NLD	Community isolate	6	0	0	0	?
E0403	1997	NLD	Community isolate	7	0	0	0	?

E0996	1998	NLD	Community isolate	47	0	0	0	?
TUH 41-67	2002	ITA	Community isolate	18	0	0	0	?
TUH 44-23	1998	NOR	Community isolate	8	0	0	0	?
TUH 44-42	1998	NOR	Community isolate	48	0	0	0	?
TUH 44-47	1999	NOR	Community isolate	246	0	0	0	?
TUH 45-05	1999	NOR	Community isolate	48	0	0	0	?
TUH 45-25	1998	NOR	Community isolate	60	0	0	0	?
Com12	2006	USA	Community isolate	107	0	0	0	?
E1485	2000	ESP	Community isolate	101	0	0	0	?
E2364	1997	DEU	Community isolate	21	0	0	0	?
E4179	2003	FRA	Community isolate	50	0	0	0	?
E4181	2003	FRA	Community isolate	17	0	0	0	?
E4182	2003	FRA	Community isolate	29	0	0	0	?
E4344	2003	FRA	Community isolate	623	0	0	0	?
E4345	2003	FRA	Community isolate	1179	0	0	0	?
E4348	2003	FRA	Community isolate	130	0	0	0	?
E4349	2004	FRA	Community isolate	696	0	0	0	?
E4351	2004	FRA	Community isolate	39	0	0	0	?
E4353	2004	FRA	Community isolate	32	0	0	0	?
E4354	2004	FRA	Community isolate	130	0	0	0	?
E4356	2004	FRA	Community isolate	130	0	0	0	?
E4357	2004	FRA	Community isolate	648	0	0	0	?
E4355	2004	FRA	Community isolate	627	0	0	0	?
E4346	2003	FRA	Community isolate	1178	0	0	0	?
E4178	2003	FRA	Community isolate	272	0	0	0	?
E4347	2003	FRA	Community isolate	1174	0	0	0	?
E4352	2004	FRA	Community isolate	92	0	0	0	?
E1046	1998	NLD	Community isolate	98	0	0	0	None
E0097	1997	NLD	Community isolate	92	0	0	0	None
E0098	1997	NLD	Community isolate	19	0	0	0	None
E0100	1997	NLD	Community isolate	1123	0	0	0	None
E0101	1997	NLD	Community isolate	1124	0	0	0	None
E0107	1997	NLD	Community isolate	995	0	0	0	None
E0110	1997	NLD	Community isolate	1182	0	0	0	None
E0114	1997	NLD	Community isolate	1125	0	0	0	None
E0116	1997	NLD	Community isolate	773	0	0	0	None
E1738	2002	ESP	Community isolate	18	0	0	0	None
E0397	1998	NLD	Community isolate	29	0	0	0	None
E0975	1998	NLD	Community isolate	130	0	0	0	None
E0982	1998	NLD	Community isolate	512	0	0	0	None
E0985	1998	NLD	Community isolate	118	0	0	0	None
E0986	1998	NLD	Community isolate	94	0	0	0	None
E0990	1998	NLD	Community isolate	1137	0	0	0	None
E1764	1996	BEL	Community isolate	6	0	0	0	None
E1012	1998	NLD	Community isolate	95	0	0	0	None
E1054	1998	NLD	Community isolate	1139	0	0	0	None
E1025	1998	NLD	Community isolate	5	0	0	0	None
E1036	1998	NLD	Community isolate	97	0	0	0	None

E1037	1998	NLD	Community isolate	97	0	0	0	None
E1327	1998	NLD	Community isolate	118	0	0	0	None
E1329	1998	NLD	Community isolate	59	0	0	0	None
E1333	1998	NLD	Community isolate	61	0	0	0	None
E1334	1998	NLD	Community isolate	21	0	0	0	None
E1766	1996	BEL	Community isolate	136	0	0	0	None
E1489	2000	ESP	Community isolate	102	0	0	0	None
E1583	2001	BEL	Community isolate	161	0	0	0	None
E8461	2014	NLD	Community isolate	650	0	0	0	None
E8511	2014	NLD	Community isolate	18	0	0	0	None
E9076	2015	NLD	Community isolate	1146	0	0	0	None
E8516	2014	NLD	Community isolate	18	0	0	0	None
E8526	2014	NLD	Community isolate	18	0	0	0	None
E8857	2015	NLD	Community isolate	266	0	0	0	None
E8862	2015	NLD	Community isolate	266	0	0	0	None
E8624	2015	NLD	Community isolate	168	0	0	0	None
E9081	2015	NLD	Community isolate	598	0	0	0	None
E8645	2015	NLD	Community isolate	168	0	0	0	None
E8665	2015	NLD	Community isolate	18	0	0	0	None
E8670	2015	NLD	Community isolate	885	0	0	0	None
E8671	2015	NLD	Community isolate	39	0	0	0	None
E8827	2015	NLD	Community isolate	1097	0	0	0	None
E8705	2015	NLD	Community isolate	1092	0	0	0	None
E8740	2015	NLD	Community isolate	266	0	0	0	None
E8745	2015	NLD	Community isolate	19	0	0	0	None
E8750	2015	NLD	Community isolate	323	0	0	0	None
E9096	2015	NLD	Community isolate	264	0	0	0	None
E8947	2015	NLD	Community isolate	264	0	0	0	None
E8952	2015	NLD	Community isolate	264	0	0	0	None
E9036	2015	NLD	Community isolate	271	0	0	0	None
E8962	2015	NLD	Community isolate	1098	0	0	0	None
E9031	2015	NLD	Community isolate	1145	0	0	0	None
E9026	2015	NLD	Community isolate	598	0	0	0	None
E9156	2015	NLD	Community isolate	1150	0	0	0	None
E9161	2015	NLD	Community isolate	598	0	0	0	None
E9256	2015	NLD	Community isolate	271	0	0	0	None
E9261	2015	NLD	Community isolate	18	0	0	0	None
E9272	2016	NLD	Community isolate	264	0	0	0	None
E9277	2016	NLD	Community isolate	1194	0	0	0	None
E1002	1998	NLD	Community isolate	54	0	0	0	None
E1028	1998	NLD	Community isolate	56	0	0	0	None
E1043	1998	NLD	Community isolate	32	0	0	0	None
E1488a	2000	ESP	Community isolate	32	0	0	0	None
E1488b	2000	ESP	Community isolate	32	0	0	0	None
E8584	2014	NLD	Community isolate	1120	0	0	0	None
E8604	2014	NLD	Community isolate	1121	0	0	0	None
E8702	2015	NLD	Community isolate	18	0	0	0	None
E8735	2015	NLD	Community isolate	18	0	0	0	None

E9111	2015	NLD	Community isolate	1148	0	0	0	None
E9121	2015	NLD	Community isolate	117	0	0	0	None
E0103	1997	NLD	Community isolate	178	0	0	0	None
E0109	1997	NLD	Community isolate	345	0	0	0	None
E8710	2015	NLD	Community isolate	296	0	0	0	None
E9106	2015	NLD	Community isolate	296	0	0	0	None
E8821	2015	NLD	Community isolate	49	0	0	0	None
E0999	1998	NLD	Community isolate	636	0	0	0	None
E0988	1998	NLD	Community isolate	1136	0	0	0	None
E1031	1998	NLD	Community isolate	96	0	0	0	None
E0996	1998	NLD	Community isolate	47	0	0	0	None
E1328	1998	NLD	Community isolate	47	0	0	0	None
E1051	1998	NLD	Community isolate	1138	0	0	0	None
E9101	2015	NLD	Community isolate	1147	0	0	0	None
E1582	2001	BEL	Community isolate	59	0	0	0	None
E0070	1997	NLD	Community isolate	374	0	0	0	*vanA*
E0066	1997	NLD	Community isolate	6	0	0	0	*vanA*
E0060	1996	NLD	Community isolate	147	0	0	0	*vanA*
E0092	1997	NLD	Community isolate	6	0	0	0	*vanA*
E0059	1996	NLD	Community isolate	1122	0	0	0	*vanA*
E0084	1997	NLD	Community isolate	5	0	0	0	*vanA*
E0129	1996	NLD	Community isolate	6	0	0	0	*vanA*
E0131	1996	NLD	Community isolate	5	0	0	0	*vanA*
E0135	1996	NLD	Community isolate	6	0	0	0	*vanA*
E0138	1996	NLD	Community isolate	6	0	0	0	*vanA*
E0139	1996	NLD	Community isolate	6	0	0	0	*vanA*
E0227	1996	NLD	Community isolate	6	0	0	0	*vanA*
E0664	1999	NLD	Community isolate	6	0	0	0	*vanA*
E0665	1999	NLD	Community isolate	6	0	0	0	*vanA*
E0666	1999	NLD	Community isolate	44	0	0	0	*vanA*
E0667	1999	NLD	Community isolate	6	0	0	0	*vanA*
E0668	1999	NLD	Community isolate	6	0	0	0	*vanA*
E0669	1999	NLD	Community isolate	1134	0	0	0	*vanA*
E0670	1999	NLD	Community isolate	217	0	0	0	*vanA*
E0671	1999	NLD	Community isolate	5	0	0	0	*vanA*
E0673	1999	NLD	Community isolate	6	0	0	0	*vanA*
E0742	1998	NLD	Community isolate	6	0	0	0	*vanA*
E0743	1998	NLD	Community isolate	139	0	0	0	*vanA*
E0744	1998	NLD	Community isolate	6	0	0	0	*vanA*
E0746	1998	NLD	Community isolate	6	0	0	0	*vanA*
E1768	1996	BEL	Community isolate	137	0	0	0	*vanA*
E9091	2015	NLD	Community isolate	5	0	0	0	*vanA*
E0088	1997	NLD	Community isolate	25	0	0	0	*vanA*
E0128	1996	NLD	Community isolate	82	0	0	0	*vanA*
E0672	1999	NLD	Community isolate	133	0	0	0	*vanA*
E0069	1997	NLD	Community isolate	6	0	0	0	*vanA*
E0130	1996	NLD	Community isolate	6	0	0	0	*vanA*
E0979	1998	NLD	Community isolate	1135	0	0	0	vanC1

20575	2008	NOR	Blood culture isolate	17	0	0	0	?
40648	2008	NOR	Blood culture isolate	18	0	0	0	?
43134	2008	NOR	Blood culture isolate	32	0	0	0	?
64750	2008	NOR	Blood culture isolate	533	0	0	0	?
81163	2008	NOR	Blood culture isolate	282	0	0	0	?
87205	2008	NOR	Blood culture isolate	580	0	0	0	?
89332	2008	NOR	Blood culture isolate	440	0	0	0	?
115920	2008	NOR	Blood culture isolate	94	0	0	0	?
129766	2008	NOR	Blood culture isolate	32	0	0	0	?
506120	2008	NOR	Blood culture isolate	579	0	0	0	?
653294	2008	NOR	Blood culture isolate	202	0	0	0	?
10146163	2008	NOR	Blood culture isolate	18	0	0	0	?
10181971	2008	NOR	Blood culture isolate	576	0	0	0	?
10397371	2008	NOR	Blood culture isolate	132	0	0	0	?
50150755	2008	NOR	Blood culture isolate	440	0	0	0	?
50183388	2008	NOR	Blood culture isolate	18	0	0	0	?
50708930	2012	NOR	Blood culture isolate	117	0	0	0	?
908301850	2008	NOR	Blood culture isolate	52	0	0	0	?
908900774	2008	NOR	Blood culture isolate	202	0	0	0	?
1410811268	2008	NOR	Blood culture isolate	202	0	0	0	?
1410820462	2008	NOR	Blood culture isolate	22	0	0	0	?
1410825114	2008	NOR	Blood culture isolate	18	0	0	0	?
10454581	2008	NOR	Blood culture isolate	279	0	0	0	?
17147	2008	NOR	Blood culture isolate	202	0	0	0	?
18317	2008	NOR	Blood culture isolate	22	0	0	0	?
18580	2008	NOR	Blood culture isolate	581	0	0	0	?
21864	2008	NOR	Blood culture isolate	574	0	0	0	?
26495	2008	NOR	Blood culture isolate	202	0	0	0	?
27719	2008	NOR	Blood culture isolate	18	0	0	0	?
31229	2008	NOR	Blood culture isolate	19	0	0	0	?
36488	2008	NOR	Blood culture isolate	296	0	0	0	?
E1463/A5FLR(vre)	1998	ESP	Blood culture isolate	17	0	0	0	?
et10	2008	NOR	Blood culture isolate	94	0	0	0	?
O2T878	1998	SWE	Blood culture isolate	17	0	0	0	?
U0218/Q1825/770402 I	1997	NLD	Blood culture isolate	17	0	0	0	?
U0229/O0160/506619	1995	NLD	Blood culture isolate	17	0	0	0	?
UW6847	2006	DEU	Blood culture isolate	78	0	0	0	?
UW6880	2006	DEU	Blood culture isolate	78	0	0	0	?
UW6900	2005	DEU	Blood culture isolate	17	0	0	0	?
E6073	2009	CHE	Blood culture isolate	78	0	0	0	?
E6076	2009	CHE	Blood culture isolate	78	0	0	0	?
E3160	2005	NLD	Blood culture isolate	117	0	0	0	?
E6072	2009	CHE	Blood culture isolate	78	0	0	0	?
E6691	2010	CHE	Blood culture isolate	18	0	0	0	?
E6074	2009	CHE	Blood culture isolate	18	0	0	0	?
E5189	2009	CHE	Blood culture isolate	22	0	0	0	?
E5190	2009	CHE	Blood culture isolate	1188	0	0	0	?

E6687	2010	CHE	Blood culture isolate	22	0	0	0	?
E6684	2010	CHE	Blood culture isolate	117	0	0	0	?
E6849	2010	CHE	Blood culture isolate	130	0	0	0	?
E6850	2010	CHE	Blood culture isolate	78	0	0	0	?
E6853	2010	CHE	Blood culture isolate	192	0	0	0	?
E6854	2010	CHE	Blood culture isolate	192	0	0	0	?
E6855	2010	CHE	Blood culture isolate	78	0	0	0	?
E6856	2010	CHE	Blood culture isolate	117	0	0	0	?
E7553	2012	NLD	Blood culture isolate	117	0	0	0	?
E6688	2010	CHE	Blood culture isolate	25	0	0	0	?
E6851	2010	CHE	Blood culture isolate	25	0	0	0	?
E7272	0	GRC	Blood culture isolate	723	0	0	0	?
E6858	2010	CHE	Blood culture isolate	994	0	0	0	?
E6693	2010	CHE	Blood culture isolate	78	0	0	0	?
E6094	2007	DEU	Blood culture isolate	117	0	0	0	None
E1250	?	CHE	Blood culture isolate	25	0	0	0	None
E1267	?	AUT	Blood culture isolate	78	0	0	0	None
E1293	2000	ITA	Blood culture isolate	50	0	0	0	None
E1186	?	DEU	Blood culture isolate	78	0	0	0	None
E1181	?	AUT	Blood culture isolate	78	0	0	0	None
E1303	?	POL	Blood culture isolate	17	0	0	0	None
E3449	2006	NLD	Blood culture isolate	117	0	0	0	None
E1721	2001	TZA	Blood culture isolate	169	0	0	0	None
E1277	?	DEU	Blood culture isolate	78	0	0	0	None
E1469	2000	ESP	Blood culture isolate	18	0	0	0	None
E1302	?	POL	Blood culture isolate	17	0	0	0	None
E7481	2012	NLD	Blood culture isolate	290	0	0	0	*vanA*
E7585	2012	NLD	Blood culture isolate	203	0	0	0	*vanA*
E7311	2012	NLD	Blood culture isolate	494	0	0	0	*vanA*
E7482	2012	NLD	Blood culture isolate	290	0	0	0	*vanA*
E7501	2012	NLD	Blood culture isolate	203	0	0	0	*vanA*
E7603	2013	NLD	Blood culture isolate	203	0	0	0	*vanA*
E7942	2013	NLD	Blood culture isolate	203	0	0	0	*vanA*
E9312	2002-03	PRT	Blood culture isolate	18	0	0	0	*vanA*
E9316	1999-2001	PRT	Blood culture isolate	132	0	0	0	*vanA*
E9335	1999	ESP	Blood culture isolate	18	0	0	0	*vanA*
E8150	2014	NLD	Blood culture isolate	18	0	0	0	*vanA*
E7195	2010	PRT	Blood culture isolate	18	0	0	0	*vanA*
E7394	2012	NLD	Blood culture isolate	730	0	0	0	*vanA*
E1435	1999	GRC	Blood culture isolate	65	0	0	0	*vanA*
E9331	2006	DEU	Blood culture isolate	202	0	0	0	*vanA*
E7300	0	GRC	Blood culture isolate	552	0	0	0	*vanA*
E8236	2015	NLD	Blood culture isolate	18	0	0	0	*vanA*
E9000	2015	NLD	Blood culture isolate	203	0	0	0	*vanA*
E9286	2005	ESP	Blood culture isolate	17	0	0	0	*vanB*
E2359	2004	SGP	Blood culture isolate	280	0	0	0	*vanB*
E2365	2004	HUN	Blood culture isolate	80	0	0	0	*vanB*
E9301	2002	ESP	Blood culture isolate	17	0	0	0	*vanB*

E9332	2004	ESP	Blood culture isolate	18	0	0	0	*vanB*
E9297	2002	ITA	Blood culture isolate	209	0	0	0	*vanB*
E7660	2013	NLD	Blood culture isolate	117	0	0	0	*vanB*

Table S2: Strains and plasmids used for laboratory experiments

	Relevant characteristic	Reference or source
***E. coli* strains**		
Rosetta Gami(DE3)*pLysS*	*E. coli* host strain for heterologous protein expression	Novagen
DH5α	*E. coli* host strain for routine cloning	Invitrogen
EC1000	*E. coli* host strain for routine cloning	Lab stock, as in [1]
OneShot Top10	*E. coli* host strain for routine cloning	Invitrogen
***E. faecium* strains**		
E1162	Clinical isolate (bloodstream)	As described in [2]
E1162Δ*tirE*	Markerless mutant of LocusTags EfmE1162_2149 - EfmE1162_2151	This study
K60-39	Clinical isolate (bloodstream)	This study
DO	Clinical isolate (bloodstream)	As described in [3]
**Plasmids**		
pRSETB- His_6	His_6-tag vector for protein Expression, Amp^r	Invitrogen, Modified as in [4]
pWS3	Gram-positive thermosensitive origin; Spc^r	As described in [5]
pDELtir	pWS3 derivative carrying genes LocusTags EfmE1162_2149 - EfmE1162_2151	This study
pEF25	GFP vector	As described in [6]

[1] K. Leenhouts, G. Buist, A. Bolhuis, A. ten Berge, J. Kiel, I. Mierau, M. Dabrowska, G. Venema, J. Kok, A general system for generating unlabelled gene replacements in bacterial chromosomes, Mol. Gen. Genet. MGG. 253 (1996) 217–224.

[2] W. van Schaik, J. Top, D.R. Riley, J. Boekhorst, J.E. Vrijenhoek, C.M. Schapendonk, A.P. Hendrickx, I.J. Nijman, M.J. Bonten, H. Tettelin, R.J. Willems, Pyrosequencing-based comparative genome analysis of the nosocomial pathogen Enterococcus faecium and identification of a large transferable pathogenicity island, BMC Genomics. 11 (2010) 239. doi:10.1186/1471-2164-11-239.

[3] X. Qin, J.R. Galloway-Peña, J. Sillanpaa, J.H. Roh, S.R. Nallapareddy, S. Chowdhury, A. Bourgogne, T. Choudhury, D.M. Muzny, C.J. Buhay, Y. Ding, S. Dugan-Rocha, W. Liu, C. Kovar, E. Sodergren, S. Highlander, J.F. Petrosino, K.C. Worley, R.A. Gibbs, G.M. Weinstock, B.E. Murray, Complete genome sequence of Enterococcus faecium strain TX16 and comparative genomic analysis of Enterococcus faecium genomes, BMC Microbiol. 12 (2012) 135. doi:10.1186/1471-2180-12-135.

[4] B.W. Bardoel, R. Vos, T. Bouman, P.C. Aerts, J. Bestebroer, E.G. Huizinga, T.H.C. Brondijk, J.A.G. van Strijp, C.J.C. de Haas, Evasion of Toll-like receptor 2 activation by staphylococcal superantigen-like protein 3, J. Mol. Med. 90 (2012) 1109–1120. doi:10.1007/s00109-012-0926-8.

[5] X. Zhang, F.L. Paganelli, D. Bierschenk, A. Kuipers, M.J.M. Bonten, R.J.L. Willems, W. van Schaik, Genome-Wide Identification of Ampicillin Resistance Determinants in Enterococcus faecium, PLOS Genet. 8 (2012) e1002804. doi:10.1371/journal.pgen.1002804.

[6] J. Top, (2018).

Table S3: Primers used in study

**Prevalence screening**	5' - 3' nucleotide sequence
TirE1-F	ACCTATCAGCACCACAGT
TirE1-R	GGACTTTGCGAGGAACAA
Hp1-F	TCCAAGTCCGTAACCTGATA
Hp1-R	CGGGAAGTGCTGTTGATT
TirE2-F	CATCCTCGGTGCTCATTT
TirE2-R	CTGACGACAGCTTCAATAGT
**Junction PCR**	
J2148_2149_F	GATATGATGGCTCATCAGTAA
J2148_2149_R	CATACAAAAAAGGTAAATAA
J2149_2150_F	CTAAAAAAAACCCTTCTCACCAT
J2149_2150_R	GTATTCAGGAGGATTAA
J2150_2151_F	ATTAAAACTTTCTTATCCAT
J2150_2151_R	CCATTTTCACTAATCTCTAA
J2151_2152_F	CATATGCCTTATACAA
J2151_2152_R	GAAGATTTGCTTCAATGA
J2152_2153_F	CCTCTTTCAAGAATAGCAC
J2152_2153_R	GATTGCAAATTTCTTCAA
**E1162ΔTIR construction**	
TirE1-F-down-EcoR1	TTAGATTTTCA GAATTC ATATTAATCCTCCTGAA
TirE2-R-up-EcoR1	TTATCTGAAC GAATTC CTAATCAATTTACTGAATCG
TirE1-check-up	GCTAGAACCCTTATA
TirE2-check-down	CCCGCTTATAGTTCC
Gblock sequence	CCTTGGCCCGGGAGAGGAAAGAATCAGGCTTTATAGAGATATTTCATCTAACAAA<br>TTACTTTAGACTGACAGTATATATCAGCAGGTACTGTCATCGGGAACGTAGCCAA<br>TATCGTATCCAAAACAGCAGGTAGAGAAGCTATTTGAGGATTCAAGTTATTCTAT<br>TTCAACCATGAAGTTAAGTTCATTAAAAATAAAGATAGTTGGTATTTCAATAATTT<br>TTGTATAGAGTTAATCACTTTATTGATGCTAATTCATCTTGATTGGTATTATAATC<br>GTATTCAAGTTGCGTGGCGTGCTTTTGCTAGAAAATATATAACTGTTCTTTATAAA<br>GAAATATAAGGATTAAACATTTTTTATCATAGAGTTTTACTTATCTATAGAATTAT<br>GTCTGTTCCTAATTCAGTTTGATGAATTAACTGATAAAAAAATATCTACTATTTAA<br>AGATACAGGGATAAGTAAGTAAAAAAATAACATGTCATTAGATAGTAGACAGTT<br>CCAACTCTTTATTGAATTCCAAGGATATCCTCCATTCTTGTATTTTGATCCACAATT<br>TATTAGGCAACATAACAGTATACCCTATCGTTACATATCCCATTCATTATTATACC<br>AGTATTTCTTCGTGACACATAAGGGTGTAACATAATAATTTCAAACGAAATGACT<br>ACAAGTAGTCATTGAAGCAAATCTTCATAAGACACCTGTAGAATATCTTTGATGG<br>CTTTCAATTGATCCAACTTAATGTGTTGGCGGCCACCTTCAATCTTAACCAACGTT<br>TCCCTAGTTATAGCAATTTTACGAAGCTGTAGGTCTCTAATCAGCTCGGTTTGACC<br>AATTCCTTTTTCTAATCGGACTCGTCTAATATTGCTACCGATTACATTTTCTTTATC

	AATAATTTTTTCCTCTTTCAAGAATAGCACACCTTTTGGACTAAAATTAGTCCTTTTTCTTATTAATCTTAAAGATTCGGTATGCTACAATGGGACTAGTATTAGTCCATATTCAATTTTAAGGAGGCTCGAGATACTG
**Expression**	
BamHI-Tir1-Fw	CGGGATCCATGGTGAGAAGGGTTTTTTTTAG
NotI-Tir1-Rv	ATATGCGGCCGCTTATTTACCTTTTTTGTATGCAC
BamHI-Hp1-Fw	CGGGATCCATGGATAAGAAAGTTTTAATAAAG
NotI-Hp1-Rv	ATATGCGGCCGCTTAATCCTCCTGAATAC
BamHI-Tir2-Fw	CGGGATCCATGTTGTATAAGGCATATGAATTGG
NotI-Tir2-Rv	ATATGCGGCCGCTTAGAGATTAGTGAAAATGGTGTTT

Table S4: Metadata of *E. faecium* genomes harbouring *tirE1-hp1-tirE2* genes.

Assembly Accession	Organism Name	BioProject Accession	BioSample Accession	Isolation Source	Geographic Location	Host
GCA_000148025.1	*Enterococcus faecium* TX0082	PRJNA47193	SAMN00189133	blood	n/a	Human
GCA_000157535.1	*Enterococcus faecium* 1,231,502	PRJNA32955	SAMN02595268	clinical	n/a	Human
GCA_000166815.1	*Enterococcus faecium* DO	PRJNA71	SAMN02440386	blood, endocarditis	USA, Houston, Texas	Human
GCA_000172675.1	*Enterococcus faecium* E1162	PRJNA29921	SAMN02471503	blood	France	Human
GCA_000172915.1	*Enterococcus faecium* U0317	PRJNA30675	SAMN02471515	urinary tract	the Netherlands, Utrecht	Human
GCA_000174395.2	*Enterococcus faecium* DO	PRJNA30627	SAMN00002237	blood	USA, Houston, Texas	Human
GCA_000239115.2	*Enterococcus faecium* E4452	PRJNA60873	SAMN02471474	dog	Netherlands, The Hague	Dog
GCA_000294835.1	*Enterococcus faecium* S447	PRJNA82069	SAMN02299515	urogenital tract	n/a	Human
GCA_000294915.1	*Enterococcus faecium* R496	PRJNA82061	SAMN02299511	blood	USA, Texas	Human
GCA_000294955.1	*Enterococcus faecium* R446	PRJNA82057	SAMN02299509	blood	USA, Texas	Human
GCA_000294975.1	*Enterococcus faecium* P1986	PRJNA82055	SAMN02299508	blood	n/a	Human
GCA_000295015.1	*Enterococcus faecium* P1140	PRJNA82051	SAMN02299506	n/a	n/a	Human
GCA_000295075.1	*Enterococcus faecium* ERV168	PRJNA82035	SAMN02299498	skin	n/a	Human
GCA_000295235.1	*Enterococcus faecium* ERV165	PRJNA82033	SAMN02299497	gastrointestinal tract	n/a	Human
GCA_000295255.1	*Enterococcus faecium* ERV161	PRJNA82031	SAMN02299496	blood	n/a	Human
GCA_000295355.1	*Enterococcus faecium* ERV102	PRJNA82029	SAMN02299495	oral cavity	n/a	Human
GCA_000295395.1	*Enterococcus faecium* C621	PRJNA82021	SAMN02299491	n/a	n/a	Human
GCA_000295455.1	*Enterococcus faecium* E422	PRJNA82025	SAMN02299493	urogenital tract	n/a	Human
GCA_000321685.1	*Enterococcus faecium* E1185	PRJNA82433	SAMN00779803	blood	France	Human
GCA_000322365.1	*Enterococcus faecium* E1644	PRJNA82513	SAMN00779847	hospitalized patient	Germany	Human
GCA_000322385.1	*Enterococcus faecium* E2369	PRJNA82515	SAMN00779848	n/a	n/a	Human
GCA_000322405.1	*Enterococcus faecium* E2560	PRJNA82517	SAMN00779849	blood	Netherlands	Human
GCA_000322445.1	*Enterococcus faecium* E6012	PRJNA82521	SAMN00779851	hospitalized patient	Latvia	Human
GCA_000322465.1	*Enterococcus faecium* E6045	PRJNA82523	SAMN00779852	hospitalized patient	Portugal	Human
GCA_000328425.1	*Enterococcus faecium* C309	PRJNA160211	SAMN02470931	peritoneal drainage fluids	China	Human
GCA_000393775.1	*Enterococcus faecium* UAA1484	PRJNA88829	SAMN00809104	n/a	n/a	Human
GCA_000394575.1	*Enterococcus faecium* UAA949	PRJNA89009	SAMN00809200	n/a	USA, New York	Human
GCA_000394595.1	*Enterococcus faecium* UAA950	PRJNA89011	SAMN00809201	n/a	USA, New York	Human
GCA_000395645.1	*Enterococcus faecium* UAA725	PRJNA88355	SAMN00808995	n/a	France, Hospital Saint-Louis, Paris	Human
GCA_000395665.1	*Enterococcus faecium* UAA825	PRJNA88357	SAMN00808996	n/a	France, Centre Hospitalier Lyon-Sud	Human
GCA_000395825.1	*Enterococcus faecium* VRE 108	PRJNA88373	SAMN00809005	n/a	Denmark	Human
GCA_000395865.1	*Enterococcus faecium* VRE 13	PRJNA88377	SAMN00809007	n/a	Denmark	Human
GCA_000395885.1	*Enterococcus faecium* VRE 84	PRJNA88379	SAMN00809008	n/a	Denmark	Human
GCA_000396925.1	*Enterococcus faecium* UAA951	PRJNA88805	SAMN00809130	n/a	USA, New York	Human

GCA_000477595.1	*Enterococcus faecium* E1165	PRJNA192894	SAMN02471488	wound	Italy, Genoa	Human
GCA_000632295.1	*Enterococcus faecium* VRE0576	PRJNA230770	SAMN02567742	blood	Sweden, Stockholm	Human
GCA_000632315.1	*Enterococcus faecium* VRE1044	PRJNA230770	SAMN02567768	blood	Sweden, Stockholm	Human
GCA_000632335.1	*Enterococcus faecium* VRE1261	PRJNA230770	SAMN02567769	blood	Sweden, Stockholm	Human
GCA_000632355.1	*Enterococcus faecium* VSE1036	PRJNA230770	SAMN02567759	blood	Sweden, Stockholm	Human
GCA_000764975.1	*Enterococcus faecium* UC7266	PRJNA261269	SAMN03070074	n/a	Italy	Human
GCA_000772505.1	*Enterococcus faecium* VRE3	PRJNA264849	SAMN03143391	clinical sample, bacteremia	USA, San Antonio, Texas	Human
GCA_000981945.1	*Enterococcus faecium* MRSN 3418	PRJNA223621	SAMN03486509	Wound	USA, DC-Baltimore	Human
GCA_001005615.1	*Enterococcus faecium* VRE1558	PRJNA278440	SAMN03433963	Blood	Brazil, Sao Paulo, Hospital das Clinicas	Human
GCA_001058095 .1	*Enterococcus faecium* 712_EFCM	PRJNA224116	SAMN03197917	Urine	US, Washington UW clinical laboratory	Human
GCA_001059685 .1	*Enterococcus faecium* 964_EFCM	PRJNA267549	SAMN03198184	Fluid	US, Washington, UW clinical laboratory	Human
GCA_001059755.1	*Enterococcus faecium* 990_EFCM	PRJNA267549	SAMN03198211	Fluid	US, Washington, UW clinical laboratory	Human
GCA_001059965.1	*Enterococcus faecium* 967_EFCM	PRJNA267549	SAMN03198187	Wound	US, Washington, UW clinical laboratory	Human
GCA_001059995.1	*Enterococcus faecium* 969_EFCM	PRJNA267549	SAMN03198189	Urine, Catheterized	US, Washington, UW clinical laboratory	Human
GCA_001518735.1	*Enterococcus faecium*6E6	PRJNA308959	SAMN04418075	feces	University of Minnesota Hospital	Human
GCA_001545595.1	*Enterococcus faecium* VRE-1300937	PRJNA301929	SAMN04270930	feces	Sweden, Uppsala Akademiska Sjukhuset	Human
GCA_001545605.1	*Enterococcus faecium*VRE-1400408	PRJNA301929	SAMN04270935	feces	Sweden, Uppsala Akademiska Sjukhuset	Human
GCA_001547025.1	*Enterococcus faecium* VRE-1402991	PRJNA301929	SAMN04270955	feces	Sweden, Uppsala Akademiska Sjukhuset	Human
GCA_001547045.1	*Enterococcus faecium* VRE-1403299	PRJNA301929	SAMN04270956	feces	Sweden, Uppsala Akademiska Sjukhuset	Human
GCA_001547115.1	*Enterococcus faecium* VRE-1402435	PRJNA301929	SAMN04270950	feces	Sweden, Uppsala Akademiska Sjukhuset	Human
GCA_001547355.1	*Enterococcus faecium* U-1313438	PRJNA301929	SAMN04270924	feces	Sweden, Uppsala Akademiska Sjukhuset	Human
GCA_001547425.1	*Enterococcus faecium* VRE-1403540	PRJNA301929	SAMN04270958	feces	Sweden, Uppsala Akademiska Sjukhuset	Human
GCA_001547445.1	*Enterococcus faecium* VRE-1404192	PRJNA301929	SAMN04270960	feces	Sweden, Uppsala Akademiska Sjukhuset	Human
GCA_001563205.1	*Enterococcus faecium* 805447/07	PRJNA308579	SAMN04397503	Pleura effusion	Austria, Vienna	Human
GCA_001895905.1	*Enterococcus faecium* VRE001	PRJNA353653	SAMN06018903	Blood	US, Tennessee, St Jude Children's Research Hospital	Human
GCA_001896235.1	*Enterococcus faecium* 4686	PRJNA355170	SAMN06065846	blood	Russia, Saint-Petersburg	Human
GCA_001969545.1	*Enterococcus faecium* VREr6	PRJNA312725	SAMN04500822	Blood	Malaysia	Human
GCA_001969645.1	*Enterococcus faecium* VREr7	PRJNA312726	SAMN04500826	Urine	Malaysia	Human
GCA_900080055.1	*Enterococcus faecium*	PRJEB13267	SAMEA3935366	Blood	Germany	Human
